# Supplementary material for: Worldwide dynamic biogeography of zoonotic and anthroponotic dengue
Source: PLoS Negl Trop Dis. 2021 Jun 7;15(6):e0009496. doi: 10.1371/journal.pntd.0009496 (PMC8211191; doi:10.1371/journal.pntd.0009496)
Supplement: S1 Table — See source references in the maintext. (DOCX) [file pntd.0009496.s001.docx]

**S1 Table. Number of dengue case reports and vector occurrences considered in the analyses; and number of presences after point transference to a 7,774-km^2^ hexagons grid.** See source references in the maintext.

|  | **Points** | | **Hexagons** |
| --- | --- | --- | --- |
| Dengue cases | | | |
|  | Messina *et al.* (2019) | Various sources | Presences |
| Late 20^th^-century reports | 3923 | 0 | 556 |
| Early 21^st^-century reports | 6993 | 4495 | 2027 |
| 2018 -2019 validation data set | 0 | 3996 | 992 |
| *Aedes aegypti* | | | |
|  | Kraemer *et al*. (2015) | Various sources | Presences |
| Late 20^th^-century occurrences | 3385 | 0 | 585 |
| Early 21^st^-century occurrences | 16739 | 6117 | 1376 |
| *Aedes albopictus* | | | |
|  | Kraemer *et al*. (2015) | Various sources | Presences |
| Late 20^th^-century occurrences | 1886 | 0 | 477 |
| Early 21^st^-century occurrences | 19892 | 2757 | 1007 |
| Sylvatic vectors | | | |
|  | Various sources | | Presences |
| *Aedes niveus* | 29 | | 16 |
| *Aedes luteocephalus* | 76 | | 18 |
| *Aedes africanus* | 71 | | 27 |
| *Aedes vittatus* | 74 | | 41 |
| *Aedes polynisiensis* | 69 | | 2 |
