# Supplementary material for: Worldwide dynamic biogeography of zoonotic and anthroponotic dengue
Source: PLoS Negl Trop Dis. 2021 Jun 7;15(6):e0009496. doi: 10.1371/journal.pntd.0009496 (PMC8211191; doi:10.1371/journal.pntd.0009496)
Supplement: S2 Table — (DOCX) [file pntd.0009496.s002.docx]

**S2 Table. Literature used for georeferencing the presence of sylvatic dengue vectors**

| **Sylvatic vectors** | **Sources** |
| --- | --- |
| *Aedes polynesiensis* | *^[1][2][3][4][5][6][7][8][9][10]^* |
| *Aedes luteocephalus* | *^[11][12][13][14][15][16][17][18][19][20][20][21][22][23][24]^* |
| *Aedes africanus* | *^[25][13][26][15][16][27][19][20][22][23][24]^* |
| *Aedes vittatus* | *^[11][12][13][27][19][28][22][24][29]^* |
| *Aedes niveus* | *^[30][31][32][33][34][35][36][37][38][39][40][41][29]^* |

**References:**

1. Lardeux F, Cheffort J. Ambient temperature effects on the extrinsic incubation period of *Wuchereria bancrofti* in *Aedes polynesiensis*: Implications for filariasis transmission dynamics and distribution in French Polynesia. Med Vet Entomol. 2001;15: 167–176. doi:10.1046/j.0269-283X.2001.00305.x

2. Chambers EW, Bossin HC, Ritchie SA, Russell RC, Dobson SL. Landing response of *Aedes (Stegomyia) polynesiensis* mosquitoes to coloured targets. Med Vet Entomol. 2013;27: 332–338. doi:10.1111/j.1365-2915.2012.01065.x

3. Richard V, Paoaafaite T, Cao-Lormeau VM. Vector Competence of *Aedes aegypti* and *Aedes polynesiensis* Populations from French Polynesia for Chikungunya Virus. PLoS Negl Trop Dis. 2016;10: 1–9. doi:10.1371/journal.pntd.0004694

4. Mercer DR, Bossin H, Sang MC, O’Connor L, Dobson SL. Monitoring Temporal Abundance and Spatial Distribution of *Aedes polynesiensis* Using BG-Sentinel Traps in Neighboring Habitats on Raiatea, Society Archipelago, French Polynesia .J Med Entomol. 2012;49: 51–60. doi:10.1603/me11087

5. Bett B. Agriculture-associated diseases research at ILRI: Emerging infectious diseases. Exch Organ Behav Teach J. 2011;12: 1–4. Available: http://www.ilri.cgiar.org/handle/10568/10627

6. Hapairai LK, Sang MAC, Sinkins SP, Bossin HC. Population Studies of the Filarial Vector *Aedes* *polynesiensis* (Diptera: Culicidae) in Two Island Settings of French Polynesia J Med Entomol. 2013;50: 965–976. doi:10.1603/me12246

7. Russell RC, Webb CE, Davies N. *Aedes aegypti* (L.) and *Aedes polynesiensis* Marks (Diptera: Culicidae) in Moorea, French Polynesia: A Study of Adult Population Structures and Pathogen (*Wuchereria bancrofti* and *Dirofilaria immitis*) Infection Rates to Indicate Regional and Seasonal Epidemi. J Med Entomol. 2005;42: 1045–1056. doi:10.1093/jmedent/42.6.1045

8. Samarawickrema WA, Sone F, Kimura E, Self RF, Cummings FR, Paulson SG. The relative importance and distribution of *Aedes polynesiensis* and *Ae. aegypti* larval habitats in Samoa. Med Vet Entomol. 1993;7: 27–36.

9. Riviere F. Ecologie de *Aedes (Stegomyia) polynesiensis*, Marks, 1951 et transmission de la filariose de Bancroft en Polynésie. Universite de Paris-Sud Centre d’Orsay. 1988.

10. Suzuki T, Kutsuna S, Taniguchi S, Tajima S, Maeki T, Kato F, et al. Dengue virus exported from côte d’ivoire to Japan, June 2017. Emerg Infect Dis. 2017;23: 1758–1760. doi:10.3201/eid2310.171132

11. Diagne CT, Diallo D, Faye O, Ba Y, Faye O, Gaye A, et al. Potential of selected Senegalese *Aedes* *spp*. mosquitoes (Diptera: Culicidae) to transmit Zika virus. BMC Infect Dis. 2015;15: 2–7. doi:10.1186/s12879-015-1231-2

12. Joseph AO, Adepeju S-OI, Omosalewa OB. Distribution, abundance and diversity of mosquitoes in Akure, Ondo State, Nigeria. J Parasitol Vector Biol. 2013;5: 132–136. doi:10.5897/JPVB2013.0133

13. Diallo D, Sall AA, Buenemann M, Chen R, Faye O, Diagne CT, et al. Landscape Ecology of Sylvatic Chikungunya Virus and Mosquito Vectors in Southeastern Senegal. PLoS Negl Trop Dis. 2012;6: 1–14. doi:10.1371/journal.pntd.0001649

14. Diallo M, Thonnon J, Traore-Lamizana M, Fontenille D. Vectors of Chikungunya virus in Senegal: Current data and transmission cycles. Am J Trop Med Hyg. 1999;60: 281–286. doi:10.4269/ajtmh.1999.60.281

15. Bang YH, Bown DN, Arata AA. Ecological studies on *Aedes Africanus* (Diptera: Culicidae) and associated species in Southeastern Nigeria. J Med Entomol. 1980;17: 411–416. doi:10.1093/jmedent/17.5.411

16. Hervy JP, Legros F, Roche JC, Monteny N, Diaco B. Circulation du virus Dengue 2 dans plusieurs milieux boisés des savanes soudaniennes de la région de Bobo-Dioulasso (Burkina Faso). Cah ORSTOM Ser Ent Med Parasitol. 1984;22: 135–143.

17. Roche JC, Cordellier R, Hervy JP. Ninety-six strains of dengue 2 virus isolated from mosquitoes collected in Ivory Coast and Upper Volta. Ann Virol. 1983;134: 233–244. Available: http://www.embase.com/search/results?subaction=viewrecord&from=export&id=L13088076%5Cnhttp://sfx.hul.harvard.edu/sfx_local?sid=EMBASE&issn=02425017&id=doi:&atitle=Ninety-six+strains+of+dengue+2+virus+isolated+from+mosquitoes+collected+in+Ivory+Coast+and+U

18. Onyido A, Ezike V, Ozumba N, Nwosu E, Ikpeze O, Obiukwu M, et al. Crepuscular Man-Biting Mosquitoes Of A Tropical Zoological Garden In Enugu, South-Eastern Nigeria. Internet J Parasit Dis. 2012;4: 4–9. doi:10.5580/11a2

19. Agwu EJ, Igbinosa IB, Isaac C. Entomological assessment of yellow fever-epidemic risk indices in Benue State, Nigeria, 2010-2011. Acta Trop. 2016;161: 18–25. doi:10.1016/j.actatropica.2016.05.005

20. Ahmed UA, Sani Z. Studies of mosquitoes in Hadejia Emirate, Jigawa state, Nigeria. Book of Proceedings of the Academic Conference on Positioning Sub-Sahara Africa for Development in the new Development. 2016. pp. 1–6.

21. Bang YH, Bown DN, Onwubiko AO. Prevalence of larvae of potential yellow fever vectors in domestic water containers in south-east Nigeria. Bull World Health Organ. 1981;59: 107–114.

22. Diallo M, Ba Y, Sall AA, Diop OM, Ndione JA, Mondo M, et al. Amplification of the sylvatic cycle of dengue virus type 2, Senegal, 1999-2000: Entomologic findings and epidemiologic considerations. Emerg Infect Dis. 2003;9: 362–367. doi:10.3201/eid0903.020219

23. Anosike JC, Nwoke BEB, Okere AN, Oku EE, Asor JE, Emmy-Egbe IO, et al. Epidemiology of tree-hole breeding mosquitoes in the tropical rainforest of Imo State, South-East Nigeria. Pediatr Dev Pathol. 1998;1: 200–209. doi:10.1007/s100249900027

24. Guindo-Coulibaly N, Adja AM, Koudou BG, Konan YL, Diallo M, Koné AB, et al. Distribution and seasonal variation of *Aedes aegypti* in the health district of Abidjan (Côte d’Ivoire). Eur J Sci Res. 2010;40: 522–530.

25. Bang YH, Knudsen AB, Onwubiko AO, Bown DN. Seasonal survival of *Aedes africanus* (Diptera: Culicidae) in Nigeria. J Med Entomol. 1983;20: 128–133. doi:10.1093/jmedent/20.2.128

26. McCrae AWR, Kirya BG. Yellow fever and Zika virus epizootics and enzootics in Uganda. Trans R Soc Trop Med Hyg. 1982;76: 552–562.

27. Cordellier R, Botjchité B, Roche J-C, Monteny N. Circulation selvatique du virus dengue 2 en 1980, dans les savanes sub-soudaniennes de Côte d’Ivoire. Ent med Parasitol. 1983;21: 165–179. Available: https://core.ac.uk/download/pdf/39874081.pdf

28. Darsie F., Richard Pradhan SP, Vaidya RG. Notes on the Mosquitoes of Nepal I . New Country Records and Revised *Aedes* Keys (Diptera, Culicidae). Seta. 1991;23: 39–45.

29. Harinasuta C, Sucharit S, Deesin T, Surathin K, Vutikes S. Bancroftian filariasis in Thailand, a new endemic area. J Trop Med Pub Hlth Seas. 1970;1: 233–245.

30. Young KI, Mundis S, Widen SG, Wood TG, Tesh RB, Cardosa J, et al. Abundance and distribution of sylvatic dengue virus vectors in three different land cover types in Sarawak, Malaysian Borneo. Parasites and Vectors. 2017;10: 1–14. doi:10.1186/s13071-017-2341-z

31. Wiwatanaratanabutr I. Geographic distribution of wolbachial infections in mosquitoes from Thailand. J Invertebr Pathol. 2013;114: 337–340. doi:10.1016/j.jip.2013.04.011

32. Santiago ATA, Claveria FG. Medically important mosquitoes (Diptera: Culicidae) identified in rural Barangay Binubusan, Lian, Batangas Province, Philippines. Philipp J Sci. 2012;141: 103–109.

33. Rogozi E, Ahmad RB, Ismail Z. Distribution and species composition of mosquitoes in three malay recreational parks. Burazeri G, Kakarriqi E, editors. Albanian Med J. 2012;4: 42–55. Available: http://www.ishp.gov.al/wp-content/uploads/2012/12/revista_nr_4_2012.pdf#page=30

34. Parker OS, Chaney AH. *Liomys irroratus* (Rodentia: Heteromyidae), a new host for *Cuterebra fontinella* (Diptera: Cuterebridae). J Med Entomol. 1979;15: 573–576. doi:10.1093/jmedent/15.5-6.573

35. Harrison BA, Rattanarithikul R, Peyton EL, Mongkolpanya K. Taxonomic changes, revised occurrence records and notes on the Culicidae of Thailand and neighboring countries. Mosq Syst. 1990;22: 196–227. Available: http://oai.dtic.mil/oai/oai?verb=getRecord&metadataPrefix=html&identifier=ADA512869

36. Chen CD, Lee HL, Stella-Wong SP, Lau KW, Sofian-Azirun M. Container survey of mosquito breeding sites in a university campus in Kuala Lumpur, Malaysia. Dengue Bull. 2009;33: 187–193.

37. Azmi NNM, Saad AR. Spatial distribution and habitat characterization of aedes mosquito larvae in Dengue hotspot areas on Penang Island. The 3rd International PSU-UNS Conferences on Bioscience. 2010. pp. 134–136.

38. Gould DJ, Bailey CL, Vongpradist S. Implication of forest mosquitoes in the transmission of *Wuchereria* *Bancrofti* in Thailand. Mosq News. 1982;42: 560–563.

39. Darsie F. Richard J, Gregory WC, Shreedhar PP. Notes on the mosquitoes of Nepal: III. Additional New Records in 1992 (Diptera: Culicidae). Mosq Syst. 1993;25: 186–191.

40. Richard, F. Dardsie J, Pradhan PP, Riddhi VG. Notes on the Mosquitoes of Nepal: II. New Species records from 1991 collections. Mosq Syst. 1992;24: 23–28.

41. Sucharit S, Rongsriyam Y, Deesin V, Komalamisra N, Apiwathnasorn C, Surathint K. Biology of Dengue Vectors and Their Control in Thailand. Trop Med. 1993;35: 253–257.
