# Supplementary material for: Worldwide dynamic biogeography of zoonotic and anthroponotic dengue
Source: PLoS Negl Trop Dis. 2021 Jun 7;15(6):e0009496. doi: 10.1371/journal.pntd.0009496 (PMC8211191; doi:10.1371/journal.pntd.0009496)
Supplement: S3 Table — Some variables were used only in specific models: *20th-century models; **refined 21st-century models; ***refined 21st-century vector models; ****disease models. (DOCX) [file pntd.0009496.s003.docx]

**S3 Table. Independent predictor variables considered for disease, vector, and transmission-risk modelling.** Some variables were used only in specific models: *20^th^ century models; **refined 21^st^ century models; ***refined 21^st^ century vector models; ****disease models.

| **Factor** | **Code** | **Variable** | **Source** |
| --- | --- | --- | --- |
| Climate | *Bio1* | Annual Mean Temperature | Chelsa (http://chelsa-climate.org) |
|  | *Bio5* | Max Temperature of Warmest Month |  |
|  | *Bio6* | Min Temperature of Coldest Month |  |
|  | *Bio7* | Temperature Annual Range (Bio5-Bio6) |  |
|  | *Bio12* | Annual Precipitation |  |
|  | *Bio15* | Precipitation Seasonality (Coefficient of Variation) |  |
| Human Concentration | *Pop_den* | Population density** | Administrative Centres & Populated Places shapefile at the Relational World Database II (RWDB2) updated in 2000 (http://www.fao.org/geonetwork) |
|  | *Dist_pop* | Distance to populated places |  |
| Infrastructures** | *Dist_road* | Distance to roads | Vector Map Level 0 at the Digital Chart of the World (DCW,http://worldmap.harvard.edu), updated in 2002 |
|  | *Dist_rail* | Distance to rail-roads |  |
| Livestock*** | *Buffaloes* | Density of buffaloes | FAO 2010(http://www.fao.org/livestock-systems/en/) |
|  | *Poultry* | Density of poultry |  |
|  | *Goats* | Density of small ruminants (goats) |  |
|  | *Pigs* | Density of pigs |  |
|  | *Sheep* | Density of small ruminants (sheep) |  |
|  | *Cattle* | Density of cattle |  |
| Topography | *Slope* | slope | From GTOPO30 (US Geological Survey 1996), using ArcGIS Desktop 10.3. |
|  | *Elev* | Elevation | GTOPO30 (US Geological Survey 1996). |
| Hydrography | *Dist_riv* | Distance to rivers | Global Drainage Basin Database GDBD. Released Version 1.0: May 29, 2007 (http://www.cger.nies.go.jp/db/gdbd/gdbd_index_e.html). |
| Ecoregions* | *MedFWS* | Mediterranean Forest, Woodlands and Scrub | Terrestrial Ecoregions of the World: A New Map of Life on Earth: A new global map of terrestrial ecoregions provides an innovative tool for conserving biodiversity [42] |
|  | *TrosubDBF* | Tropical and Subtropical Dry Broadleaf Forest |  |
|  | *TempCF* | Temperate Coniferous Forests |  |
|  | *TempBMF* | Temperate Broadleaf and Mixed Forests |  |
|  | *TrosubCF* | Tropical and Subtropical Coniferous Forests |  |
|  | *DeXS* | Deserts and Xeric Shrublands |  |
|  | *Mangro* | Mangroves |  |
|  | *TrosubMBF* | Tropical and Subtropical Moist Broadleaf Forest |  |
|  | *BorFT* | Boreal Forests/Taiga |  |
|  | *TrosubGSS* | Tropical and Subtropical Grasslands, Savannas and Shrublands |  |
|  | *TempGSS* | Temperate Grasslands, Savannas and Shrublands |  |
|  | *FloGS* | Flooded Grassland and Savannas |  |
|  | *MonGS* | Montane Grasslands and Shrublands |  |
|  | *Tundra* | Tundra |  |
| Agriculture | *Class 11-14* | Croplands | GlobCover (GC) Land Cover version 2.3 database for 2009 [43] |
|  | *Class 20* | Mosaic Cropland (50-70%) / Vegetation (grassland, shrubland, forest) (20-50%)** |  |
|  | *Class 30* | Mosaic Vegetation (grassland, shrubland, forest) (50-70%) / Cropland (20-50%)** |  |
|  | *Equi_irrig* | Percentage of area equipped for irrigation*** | Global Map of Irrigation Areas (version 4.0.1) around the year 2000 (http://www.fao.org/nr/water) |
| Ecosystem Types** | *Class 40* | Closed to open (>15%) broadleaved evergreen and/or semi-deciduous forest (>5m) | GlobCover (GC) Land Cover version 2.3 database for 2009 [43] |
|  | *Class 50* | Closed (>40%) broadleaved deciduous forest (>5m) |  |
|  | *Class 60* | Open (15-40%) broadleaved deciduous forest (>5m) |  |
|  | *Class 70* | Closed (>40%) needleleaved evergreen forest (>5m) |  |
|  | *Class 90* | Open (15-40%) needleleaved deciduous or evergreen forest (>5m) |  |
|  | *Class 100* | Closed to open (>15%) mixed broadleaved and needleleaved forest (>5m) |  |
|  | *Class 110* | Mosaic Forest/Shrubland (50-70%) / Grassland (20-50%) |  |
|  | *Class 120* | Mosaic Grassland (50-70%) / Forest/Shrubland (20-50%) |  |
|  | *Class 130* | Closed to open (>15%) shrubland (<5m) |  |
|  | *Class 140* | Closed to open (>15%) grassland |  |
|  | *Class 150* | Sparse (>15%) vegetation (woody vegetation, shrubs, grassland) |  |
|  | *Class 160* | Closed (>40%) broadleaved semi-deciduous and/or evergreen forest regularly flooded - Saline water |  |
|  | *Class 170* | Closed (>40%) broadleaved semi-deciduous and/or evergreen forest regularly flooded - Saline water |  |
|  | *Class 180* | Closed to open (>15%) vegetation (grassland, shrubland, woody vegetation) on regularly flooded or waterlogged soil - Fresh, brackish or saline water |  |
|  | *Class 200* | Bare areas |  |
|  | *Class 220* | Permanent snow and ice |  |
| Forest loss** | *Forest loss* | Non intact forest | High-Resolution Global Maps of 21^st^-Century Forest Cover Change [44] |
| Logit equation | *Y-20^th^ century* | 20^th^-century-model logit equation | Linear combinations of predictor variables that form part of the logistic-regression equations |
| Spatial descriptors | *Fx* | Spatial trend, where “x” represents a continent | Linear combination of spatial variables derived from continental-scale trend surface analyses [45] |
| Primate chorotypes**** | *ASx, AFy and SAz* | Chorotype species richness. *ASx*: Asian chorotype “x”; *AFy*: African chorotype “y”; *SAz*: South-American chorotype “z” | Supplementary Figs. 5-7 [46] |

**References:**

[42] Olson DM, Dinerstein E, Wikramanayake E, Burgess ND, Powell G, Underwood E, et al. Terrestrial Ecoregions of the World: A New Map of Life on Earth: A new global map of terrestrial ecoregions provides an innovative tool for conserving biodiversity. Bioscience. 2001; 51: 933–938.

[43] Bontemps S. GLOBCOVER 2009 Products Description and Validation Report. 2011.

[44] Hansen MC. High-resolution global maps of 21^st^-century forest cover change. Science*.* 2013;342: 850–853

[45] Legendre P. Spatial autocorrelation: Trouble or New Paradigm? Ecology. 1993;74: 1659–1673

[46] Olivero J, Real R, Márquez AL. Fuzzy chorotypes as a conceptual tool to improve insight into biogeographic patterns. Syst Biol. 2011;60: 645–660. doi:10.1093/sysbio/syr026
