# Supplementary material for: Worldwide dynamic biogeography of zoonotic and anthroponotic dengue
Source: PLoS Negl Trop Dis. 2021 Jun 7;15(6):e0009496. doi: 10.1371/journal.pntd.0009496 (PMC8211191; doi:10.1371/journal.pntd.0009496)
Supplement: S4 Table — Variables in bold letters are mentioned in the results section of the main text. B: variable coefficient; SE: standard error; W: Wald parameter; DF: degrees of freedom; S: statistical significance. Variable codes as in S3 Table. (DOCX) [file pntd.0009496.s004.docx]

**S4 Table.** **Vector-model (*Aedes aegypti*) logit equations** **(i.e., linear combinations of predictor variables that form part of the logistic-regression equations).** Variables in bold letters are mentioned in the results section of the main text. B: variable coefficient; SE: standard error; W: Wald parameter; DF: degrees of freedom; S: statistical significance. Variable codes as in Supplementary Table 3.

| **20^th^-century model** | | | | | | | | | | | |
| --- | --- | --- | --- | --- | --- | --- | --- | --- | --- | --- | --- |
| ***Model* goodness of fit** | χ² = 1772.271; *p*<0.05 | | | | | | | | | | |
| **Variable** | **B** | | | **SE** | **W** | | | **DF** | | | **S** |
| ***Bio12*** | 0.215x10^-3^ | | | 0.723x10^-4^ | 8.854 | | | 1 | | | 0.003 |
| ***Bio5*** | 0.568x10^-2^ | | | 0.002 | 13.581 | | | 1 | | | 0.228x10^-3^ |
| ***Dist_pop*** | -0.483x10^-4^ | | | 0.476x10^-5^ | 103.018 | | | 1 | | | 0.332x10^-23^ |
| *Elev* | -0.342x10^-3^ | | | 0.122x10^-3^ | 7.828 | | | 1 | | | 0.005 |
| ***FAmericanorth*** | 5.103 | | | 0.233 | 478.237 | | | 1 | | | 0.517x10^-105^ |
| ***FAmericasouth*** | 4.765 | | | 0.293 | 264.171 | | | 1 | | | 0.211x10^-58^ |
| ***FAfrica*** | 4.230 | | | 0.284 | 222.491 | | | 1 | | | 0.259x10^-49^ |
| ***FAsia*** | 3.953 | | | 0.262 | 228.428 | | | 1 | | | 0.131x10^-50^ |
| ***FOceania*** | 5.543 | | | 0.310 | 318.722 | | | 1 | | | 0.275x10^-70^ |
| *Constant* | -6.277 | | | 0.539 | 135.832 | | | 1 | | | 0.217x10^-30^ |
|  | | | | | | | | | | | |
| **21^st^-century model** | | | | | | | | | | | |
| ***Model* goodness of fit** | | χ² = 5016.603; *p*<0.05 | | | | | | | | | |
| **Variable** | | **B** | | **SE** | | **W** | | | **DF** | | **S** |
| *Y-20th century* | | 0.056 | | 0.053 | | 1.126 | | | 1 | | 0.289 |
| *Dist_pop* | | -0.150x10^-4^ | | 0.339x10^-5^ | | 19.530 | | | 1 | | 0.100x10^-4^ |
| ***Bio6*** | | 0.008 | | 0.001 | | 146.720 | | | 1 | | 0.904x10^-33^ |
| *Slope* | | 0.092 | | 0.024 | | 14.573 | | | 1 | | 0.135x10^-3^ |
| ***TempGSS*** | | 1.293 | | 0.262 | | 24.291 | | | 1 | | 0.828x10^-6^ |
| ***TempCF*** | | 1.163 | | 0.313 | | 13.854 | | | 1 | | 0.198x10^-3^ |
| ***TrosubDBF*** | | 0.636 | | 0.154 | | 17.078 | | | 1 | | 0.36x10^-4^ |
| ***Fafrica*** | | 1.760 | | 0.280 | | 39.436 | | | 1 | | 0.339x10^-9^ |
| ***FAsia*** | | 2.262 | | 0.247 | | 83.926 | | | 1 | | 0.514x10^-19^ |
| ***FOceania*** | | 0.966 | | 0.383 | | 6.343 | | | 1 | | 0.012 |
| ***FAmericanorth*** | | 2.485 | | 0.293 | | 71.934 | | | 1 | | 0.223x10^-16^ |
| ***FAmericasouth*** | | 6.991 | | 0.263 | | 706.823 | | | 1 | | 0.982x10^-155^ |
| ***FEurope*** | | 6.334 | | 2.431 | | 6.789 | | | 1 | | 0.009 |
| *Constant* | | -4.725 | | 0.253 | | 348.718 | | | 1 | | 0.806x10^-77^ |
|  | | | | | | | | | | | |
| **21^st^-century refined model** | | | | | | | | | | | |
| ***Model* goodness of fit** | | | χ² = 5227.031; *p*<0.05 | | | | | | | | |
| **Variable** | | | **B** | **SE** | | | **W** | | | **DF** | **S** |
| *Y-20th century* | | | 0.096 | 0.048 | | | 3.949 | | | 1 | 0.047 |
| *Bio12* | | | -0.207x10^-3^ | 0.65x10^-4^ | | | 10.235 | | | 1 | 0.001 |
| ***Bio6*** | | | 0.010 | 0.001 | | | 137.132 | | | 1 | 0.113x10^-30^ |
| *Class 110* | | | -2.715 | 0.922 | | | 8.678 | | | 1 | 0.003 |
| *Class 150* | | | -2.273 | 0.994 | | | 5.228 | | | 1 | 0.022 |
| *Class 160* | | | -2.690 | 0.886 | | | 9.211 | | | 1 | 0.002 |
| *Class 200* | | | -1.483 | 0.389 | | | 14.538 | | | 1 | 0.137x10^-3^ |
| *Class 50* | | | -1.271 | 0.404 | | | 9.899 | | | 1 | 0.002 |
| *Class 70* | | | 1.336 | 0.573 | | | 5.442 | | | 1 | 0.020 |
| *Dist_pop* | | | -0.1x10^-4^ | 0.315x10^-5^ | | | 10.161 | | | 1 | 0.001 |
| *Equi_irrig* | | | 0.015 | 0.005 | | | 9.578 | | | 1 | 0.002 |
| ***Pop_den*** | | | 0.001 | 0.158x10^-3^ | | | 69.113 | | | 1 | 0.929x10^-16^ |
| ***Poultry*** | | | 0.72x10^-4^ | 0.627x10^-4^ | | | 8.694 | | | 1 | 0.003 |
| *Sheep* | | | -0.008 | 0.003 | | | 6.286 | | | 1 | 0.012 |
| *Slope* | | | 0.111 | 0.026 | | | 18.056 | | | 1 | 0.214x10^-4^ |
| ***FAfrica*** | | | 1.071 | 0.241 | | | 19.747 | | | 1 | 0.884x10^-5^ |
| ***FAsia*** | | | 1.016 | 0.209 | | | 23.579 | | | 1 | 0.112x10^-5^ |
| ***FAmericanorth*** | | | 1.838 | 0.251 | | | 53.673 | | | 1 | 0.237x10^-12^ |
| ***FAmericasouth*** | | | 6.222 | 0.223 | | | 780.911 | | | 1 | 0.763x10^-171^ |
| ***FEurope*** | | | 6.601 | 2.613 | | | 6.380 | | | 1 | 0.012 |
| *Constant* | | | -4.034 | 0.248 | | | 263.853 | | | 1 | 0.248x10^-58^ |
