# Supplementary material for: Worldwide dynamic biogeography of zoonotic and anthroponotic dengue
Source: PLoS Negl Trop Dis. 2021 Jun 7;15(6):e0009496. doi: 10.1371/journal.pntd.0009496 (PMC8211191; doi:10.1371/journal.pntd.0009496)
Supplement: S5 Table — Variables in bold letters are mentioned in the results section of the main text. B: variable coefficient; SE: standard error; W: Wald parameter; DF: degrees of freedom; S: statistical significance. Variable codes as in S3 Table. (DOCX) [file pntd.0009496.s005.docx]

**S5 Table.** **Vector-model (*Aedes albopictus*) logit equations** **(i.e., linear combinations of predictor variables that form part of the logistic-regression equations).** Variables in bold letters are mentioned in the results section of the main text. B: variable coefficient; SE: standard error; W: Wald parameter; DF: degrees of freedom; S: statistical significance. Variable codes as in Supplementary Table 3.

| **20^th^-century model** | | | | | | | | |
| --- | --- | --- | --- | --- | --- | --- | --- | --- |
| ***Model* goodness of fit** | χ² =2714.105; *p*<0.05 | | | | | | | |
| **Variable** | **B** | | **SE** | **W** | | **DF** | | **S** |
| ***Bio12*** | 0.001 | | 0.117x10^-3^ | 20.335 | | 1 | | 0.650x10^-5^ |
| ***Bio7*** | 0.003 | | 0.001 | 8.958 | | 1 | | 0.276x10^-2^ |
| ***Dist_pop*** | -0.334x10^-4^ | | 0.653x10^-5^ | 26.154 | | 1 | | 0.315x10^-6^ |
| ***Elev*** | -0.001 | | 0.220x10^-3^ | 13.598 | | 1 | | 0.226x10^-3^ |
| ***TempCF*** | 1.242 | | 0.378 | 10.801 | | 1 | | 0.001 |
| ***FAmericanorth*** | 8.533 | | 0.371 | 529.728 | | 1 | | 0.324x10^-116^ |
| ***FAmericasouth*** | 6.204 | | 0.451 | 189.334 | | 1 | | 0.444x10^-42^ |
| ***FEurope*** | 6.186 | | 0.442 | 195.751 | | 1 | | 0.176x10^-43^ |
| ***FAfrica*** | 5.366 | | 0.533 | 101.278 | | 1 | | 0.799x10^-23^ |
| ***FAsia*** | 5.429 | | 0.458 | 140.438 | | 1 | | 0.213x10^-31^ |
| ***FOceania*** | 6.235 | | 0.538 | 134.205 | | 1 | | 0.493x10^-30^ |
| *Constant* | -7.590 | | 0.479 | 250.819 | | 1 | | 0.172x10^-56^ |
|  | | | | | | | | |
| **21^st^-century model** | | | | | | | | |
| ***Model* goodness of fit** | | χ² = 4356.324; *p*<0.05 | | | | | | |
| **Variable** | | **B** | **SE** | | **W** | **DF** | | **S** |
| *Y-20th century* | | 0.140 | 0.029 | | 22.978 | 1 | | 0.164x10^-5^ |
| ***Dist_pop*** | | -0.242x10^-4^ | 0.351x10^-5^ | | 47.670 | 1 | | 0.504x10^-11^ |
| ***Bio15*** | | 0.010 | 0.002 | | 26.120 | 1 | | 0.321x10^-6^ |
| *Bio12* | | 0.208x10^-3^ | 0.946x10^-4^ | | 4.849 | 1 | | 0.028 |
| ***Bio7*** | | -0.003 | 0.001 | | 8.259 | 1 | | 0.004 |
| ***MedFWS*** | | 1.807 | 0.269 | | 45.225 | 1 | | 0.176x10^-10^ |
| ***TempBMF*** | | 1.100 | 0.204 | | 29.149 | 1 | | 0.673x10^-7^ |
| ***TempGSS*** | | 1.175 | 0.280 | | 17.566 | 1 | | 0.278x10^-5^ |
| ***TrosubMBF*** | | 0.848 | 0.168 | | 25.577 | 1 | | 0.425x10^-7^ |
| ***FAfrica*** | | 3.225 | 0.282 | | 130.421 | 1 | | 0.332x10^-29^ |
| ***FAsia*** | | 2.785 | 0.303 | | 84.235 | 1 | | 0.439x10^-19^ |
| ***FOceania*** | | 2.168 | 0.506 | | 18.350 | 1 | | 0.184x10^-4^ |
| ***FAmericanorth*** | | 4.372 | 0.358 | | 148.809 | 1 | | 0.316x10^-33^ |
| ***FAmericasouth*** | | 7.702 | 0.248 | | 962.278 | 1 | | 0.284x10^-210^ |
| ***FEurope*** | | 5.198 | 0.321 | | 261.777 | 1 | | 0.703x10^-58^ |
| *Constant* | | -4.843 | 0.416 | | 135.328 | 1 | | 0.280x10^-30^ |
|  | | | | | | | | |
| **21^st^-century refined model** | | | | | | | | |
| ***Model* goodness of fit** | | χ² = 4520.879; *p*<0.05 | | | | | | |
| **Variable** | | **B** | **SE** | | **W** | | **DF** | **S** |
| *Y-20th century* | | 0.089 | 0.029 | | 9.124 | | 1 | 0.003 |
| ***Bio15*** | | 0.009 | 0.002 | | 20.732 | | 1 | 0.528x10^-5^ |
| *Bio12* | | 0.341x10^-3^ | 0.909x10^-4^ | | 14.081 | | 1 | 0.175x10^-3^ |
| *Class 130* | | -1.526 | 0.413 | | 13.672 | | 1 | 0.218x10^-3^ |
| *Class 200* | | -7.853 | 2.891 | | 7.382 | | 1 | 0.007 |
| *Class 40* | | 1.118 | 0.275 | | 16.548 | | 1 | 0.474x10^-4^ |
| ***Dist_rail*** | | -0.108x10^-5^ | 0.465x10^-6^ | | 5.387 | | 1 | 0.203x10^-1^ |
| ***Dist_road*** | | -0.406x10^-5^ | 0.169x10^-5^ | | 5.743 | | 1 | 0.166x10^-1^ |
| ***Dist_pop*** | | -0.135x10^-4^ | 0.359x10^-5^ | | 14.144 | | 1 | 0.169x10^-3^ |
| ***Pop_den*** | | 0.002 | 0.163x10^-3^ | | 107.589 | | 1 | 0.331x10^-24^ |
| ***Pigs*** | | 0.002 | 0.001 | | 4.435 | | 1 | 0.035 |
| ***FAfrica*** | | 3.219 | 0.281 | | 131.037 | | 1 | 0.243x10^-29^ |
| ***FAsia*** | | 2.350 | 0.325 | | 52.336 | | 1 | 0.468x10-^12^ |
| ***FOceania*** | | 3.215 | 0.569 | | 31.875 | | 1 | 0.164x10^-7^ |
| ***FAmericanorth*** | | 4.532 | 0.360 | | 158.799 | | 1 | 0.207x10^-35^ |
| ***FAmericasouth*** | | 7.891 | 0.251 | | 985.359 | | 1 | 0.273x10^-215^ |
| ***FEurope*** | | 6.099 | 0.291 | | 440.676 | | 1 | 0.771x10^-97^ |
| *Constant* | | -5.497 | 0.339 | | 263.690 | | 1 | 0.268x10^-58^ |
