# Supplementary material for: Worldwide dynamic biogeography of zoonotic and anthroponotic dengue
Source: PLoS Negl Trop Dis. 2021 Jun 7;15(6):e0009496. doi: 10.1371/journal.pntd.0009496 (PMC8211191; doi:10.1371/journal.pntd.0009496)
Supplement: S6 Table — The Aedes polynesiensis model was only based on the spatial factor. For the rest of species, an environmental model and a spatial model were intersected. These decisions responded to the geographically restricted character of these species distributions. Variables in bold letters are mentioned in the results section of the main text. B: variable coefficient; SE: standard error; W: Wald parameter; DF: degrees of freedom; S: statistical significance. Variable codes as in S3 Table. (DOCX) [file pntd.0009496.s006.docx]

**S6 Table.** **Sylvatic-vector-model logit equations** **(i.e., linear combinations of predictor variables that form part of the logistic-regression equations).** The *Aedes polynesiensis* model was only based on the spatial factor. For the rest of species, an environmental model and a spatial model were intersected. These decisions responded to the geographically restricted character of these species distributions. Variables in bold letters are mentioned in the results section of the main text. B: variable coefficient; SE: standard error; W: Wald parameter; DF: degrees of freedom; S: statistical significance. Variable codes as in Supplementary Table 3.

| ***Aedes africanus*** | | | | | |
| --- | --- | --- | --- | --- | --- |
| ***Model goodness of fit*** | χ² =78.913; *p*<0.05 | | | | |
|  |  |  |  |  |  |
| **Variable** | **B** | **SE** | **W** | **DF** | **S** |
| ***TrosubGSS*** | 3.921 | 1.581 | 6.149 | 1 | 0.013 |
| ***Bio6*** | 0.019 | 0.007 | 8.364 | 1 | 0.004 |
| ***TrosubMBF*** | 3.593 | 1.709 | 4.42 | 1 | 0.036 |
| *Bio15* | 0.017 | 0.007 | 6.285 | 1 | 0.012 |
| *Mangro* | 4.342 | 2.368 | 3.363 | 1 | 0.067 |
| *Constant* | -13.889 | 2.192 | 40.148 | 1 | 0.235X10^-9^ |
| ***Aedes luteocephalus*** | | | | | |
| ***Model goodness of fit*** | χ² = 60.025; p<0.05 | | | | |
|  |  |  |  |  |  |
| **Variable** | **B** | **SE** | **W** | **DF** | **S** |
| ***Bio6*** | 0.032 | 0.009 | 13.419 | 1 | 0.249X10^-3^ |
| ***TrosubGSS*** | 1.763 | 0.607 | 8.43 | 1 | 0.004 |
| ***Bio5*** | 0.014 | 0.007 | 3.787 | 1 | 0.052 |
| *Constant* | -17.481 | 3.542 | 24.351 | 1 | 0.803X10^-6^ |
| ***Aedes niveus*** | | | | | |
| ***Model goodness of fit*** | χ² = 47.551; p<0.05 | | | | |
|  |  |  |  |  |  |
| **Variable** | **B** | **SE** | **W** | **DF** | **S** |
| ***Bio6*** | 0.011 | 0.004 | 6.228 | 1 | 0.013 |
| *Slope* | 0.438 | 0.103 | 18.231 | 1 | 0.2X10^-4^ |
| ***TrosubMBF*** | 1.568 | 0.773 | 4.116 | 1 | 0.042 |
| *Constant* | -10.039 | 0.892 | 126.606 | 1 | 0.227X10^-28^ |
| ***Aedes vittatus*** | | | | | |
| ***Model goodness of fit*** | χ² = 91.570; p<0.05 | | | | |
|  |  |  |  |  |  |
| **Variable** | **B** | **SE** | **W** | **DF** | **S** |
| ***Bio6*** | 0.009 | 0.002 | 15.015 | 1 | 0.107X10^-3^ |
| ***Bio5*** | 0.014 | 0.003 | 15.855 | 1 | 0.68X10^-4^ |
| ***Dist_pop*** | -0.58X10^-4^ | 0.15X10^-4^ | 15.095 | 1 | 0.102X10^-3^ |
| ***MedFWS*** | 2.089 | 0.561 | 13.852 | 1 | 0.198X10^-3^ |
| *Constant* | -9.991 | 1.283 | 60.658 | 1 | 0.679X10^-14^ |
| ***Aedes polynesiensis*** | | | | | |
| ***Model goodness of fit*** | χ² = 30.295; p<0.05 | | | | |
|  |  |  |  |  |  |
| **Variable** | **B** | **SE** | **W** | **DF** | **S** |
| *Lat* | -0.842 | 48.929 | 0.296X10^-3^ | 1.000 | 0.986 |
| *Long* | -0.175 | 8.291 | 0.447X10^-3^ | 1.000 | 0.983 |
| *Constant* | -26.676 | 1143.681 | 0.001 | 1.000 | 0.981 |
