# Supplementary material for: Worldwide dynamic biogeography of zoonotic and anthroponotic dengue
Source: PLoS Negl Trop Dis. 2021 Jun 7;15(6):e0009496. doi: 10.1371/journal.pntd.0009496 (PMC8211191; doi:10.1371/journal.pntd.0009496)
Supplement: S7 Table — The Aedes polynesiensis model was only based on the spatial factor. For the rest of species, an environmental model and a spatial model were intersected. These decisions responded to the geographically restricted character of these species distributions. Variables in bold letters are mentioned in the results section of the main text. B: variable coefficient; SE: standard error; W: Wald parameter; DF: degrees of freedom; S: statistical significance. Variable codes as in S3 Table. (DOCX) [file pntd.0009496.s007.docx]

**S7 Table.** **Sylvatic-vector refined-model logit equations** **(i.e., linear combinations of predictor variables that form part of the logistic-regression equations).** The *Aedes polynesiensis* model was only based on the spatial factor. For the rest of species, an environmental model and a spatial model were intersected. These decisions responded to the geographically restricted character of these species distributions. Variables in bold letters are mentioned in the results section of the main text. B: variable coefficient; SE: standard error; W: Wald parameter; DF: degrees of freedom; S: statistical significance. Variable codes as in Supplementary Table 3.

| ***Aedes africanus*** | | | | | |
| --- | --- | --- | --- | --- | --- |
| ***Model goodness of fit*** | χ² = 130.737 ; *p*<0.05 | | | | |
|  |  |  |  |  |  |
| **Variable** | **B** | **SE** | **W** | **DF** | **S** |
| *Bio15* | 0.024 | 0.006 | 14.666 | 1 | 0.128X10^-3^ |
| *Bio12* | 0.001 | 0.249X10^-3^ | 8.88 | 1 | 0.003 |
| ***Bio6*** | 0.024 | 0.006 | 13.762 | 1 | 0.207X10^-3^ |
| *Class 130* | 2.731 | 1.19 | 5.266 | 1 | 0.022 |
| *Class 30* | 5.707 | 0.898 | 40.344 | 1 | 0.213X10^-9^ |
| *Class 60* | 6.077 | 1.053 | 33.303 | 1 | 0.789X10^-8^ |
| *Pop_den* | 0.001 | 0.337X10^-3^ | 3.035 | 1 | 0.081 |
| ***Goats*** | 0.012 | 0.004 | 8.971 | 1 | 0.003 |
| ***Sheep*** | 0.016 | 0.004 | 13.398 | 1 | 0.252X10^-3^ |
| *Constant* | -15.508 | 1.751 | 78.463 | 1 | 0.815X10^-18^ |
| ***Aedes luteocephalus*** | | | | | |
| ***Model goodness of fit*** | χ² = 105.707; p<0.05 | | | | |
|  |  |  |  |  |  |
| **Variable** | **B** | **SE** | **W** | **DF** | **S** |
| ***Bio6*** | 0.035 | 0.010 | 12.199 | 1 | 0.478X10^-3^ |
| ***Bio5*** | 0.031 | 0.009 | 12.264 | 1 | 0.462X10^-3^ |
| *Class 130* | 3.806 | 1.355 | 7.889 | 1 | 0.005 |
| *Class 30* | 6.752 | 1.236 | 29.838 | 1 | 0.470X10^-7^ |
| *Class 60* | 6.486 | 1.299 | 24.931 | 1 | 0.594X10^-6^ |
| ***Goats*** | 0.017 | 0.004 | 20.117 | 1 | 0.7X10^-5^ |
| *Pop_den* | 0.001 | 0.362X10^-3^ | 7.270 | 1 | 0.007 |
| *Constant* | -25.799 | 4.553 | 32.113 | 1 | 0.145X10^-7^ |
| ***Aedes niveus*** | | | | | |
| ***Model goodness of fit*** | χ² = 88.027; p<0.05 | | | | |
|  |  |  |  |  |  |
| **Variable** | **B** | **SE** | **W** | **DF** | **S** |
| *Bio12* | 0.001 | 0.259X10^-3^ | 7.818 | 1 | 0.005 |
| ***Bio6*** | 0.017 | 0.004 | 14.87 | 1 | 0.115X10^-3^ |
| ***Class 11-14*** | 4.736 | 1.483 | 10.204 | 1 | 0.001 |
| *Equi_irrig* | 0.054 | 0.019 | 8.377 | 1 | 0.004 |
| *Slope* | 0.687 | 0.128 | 28.791 | 1 | 0.806X10^-7^ |
| *Constant* | -13.729 | 1.485 | 85.494 | 1 | 0.232X10^-19^ |
| ***Aedes vittatus*** | | | | | |
| ***Model goodness of fit*** | χ² = 122.41 ; p<0.05 | | | | |
|  |  |  |  |  |  |
| **Variable** | **B** | **SE** | **W** | **DF** | **S** |
| ***Bio6*** | 0.012 | 0.002 | 28.833 | 1 | 0.789X10^-7^ |
| ***Buffaloes*** | 0.021 | 0.006 | 12.646 | 1 | 0.376X10^-3^ |
| *Class 130* | 2.513 | 0.754 | 11.117 | 1 | 0.001 |
| *Class 30* | 2.428 | 0.815 | 8.864 | 1 | 0.003 |
| *Dist_rail* | -0.13X10^-4^ | 0.4X10^-5^ | 12.845 | 1 | 0.338X10^-3^ |
| ***Goats*** | 0.007 | 0.003 | 7.229 | 1 | 0.007 |
| ***Sheep*** | 0.008 | 0.003 | 7.472 | 1 | 0.006 |
| *Constant* | -7.24 | 0.465 | 242.182 | 1 | 0.131X10^-53^ |
| ***Aedes polynesiensis*** | | | | | |
| ***Model goodness of fit*** | χ² = 30.295; p<0.05 | | | | |
|  |  |  |  |  |  |
| **Variable** | **B** | **SE** | **W** | **DF** | **S** |
| *Lat* | -0.842 | 48.929 | 0.296X10^-3^ | 1 | 0.986 |
| *Long* | -0.175 | 8.291 | 0.447X10^-3^ | 1 | 0.983 |
| *Constant* | -26.676 | 1143.681 | 0.001 | 1 | 0.981 |
