# Supplementary material for: Worldwide dynamic biogeography of zoonotic and anthroponotic dengue
Source: PLoS Negl Trop Dis. 2021 Jun 7;15(6):e0009496. doi: 10.1371/journal.pntd.0009496 (PMC8211191; doi:10.1371/journal.pntd.0009496)
Supplement: S8 Table — Variables in bold letters are mentioned in the results section of the main text. B: variable coefficient; SE: standard error; W: Wald parameter; DF: degrees of freedom; S: statistical significance. Variable codes as in S3 Table. (DOCX) [file pntd.0009496.s008.docx]

**S8 Table.** **Disease-model logit equations (i.e., linear combinations of predictor variables that form part of the logistic-regression equations).** Variables in bold letters are mentioned in the results section of the main text. B: variable coefficient; SE: standard error; W: Wald parameter; DF: degrees of freedom; S: statistical significance. Variable codes as in Supplementary Table 3.

| **20^th^-century model** | | | | | |
| --- | --- | --- | --- | --- | --- |
| ***Model* goodness of fit** | χ² = 2133.082; *p*<0.05 | | | | |
| **Variable** | **B** | **SE** | **W** | **DF** | **S** |
| ***Bio6*** | 0.001 | 0.001 | 0.238 | 1 | 0.626 |
| ***Dist_pop*** | -0.828x10^-4^ | 5.73x10^-5^ | 209.014 | 1 | 0.225x10^-46^ |
| ***Elev*** | -0.001 | 1.66x10^-3^ | 32.365 | 1 | 0.128x10^-7^ |
| ***FAmerica*** | 4.157 | 0.453 | 84.376 | 1 | 0.409x10^-19^ |
| ***FAfrica*** | 2.363 | 0.452 | 27.283 | 1 | 0.176x10^-6^ |
| ***FAsia-Oceania*** | 3.372 | 0.382 | 78.125 | 1 | 0.967x10^-18^ |
| ***DeXS*** | 2.920 | 0.605 | 23.262 | 1 | 0.141x10^-5^ |
| ***Mangro*** | 3.229 | 0.817 | 15.608 | 1 | 0.779x10^-4^ |
| ***MonGS*** | 3.141 | 1.209 | 6.757 | 1 | 0.009 |
| ***TrosubGSS*** | 2.950 | 0.613 | 23.196 | 1 | 0.146x10^-5^ |
| ***TrosubCF*** | 4.381 | 0.779 | 31.594 | 1 | 0.190x10^-7^ |
| ***TrosubMBF*** | 3.065 | 0.609 | 25.301 | 1 | 0.491x10^-6^ |
| ***TrosubDBF*** | 3.580 | 0.620 | 33.339 | 1 | 0.774x10^-8^ |
| ***SA4*** | 0.268 | 0.073 | 13.409 | 1 | 0.250x10^-3^ |
| ***SA5*** | 0.279 | 0.093 | 9.018 | 1 | 0.003 |
| ***SA14*** | 0.519 | 0.227 | 5.225 | 1 | 0.022 |
| ***AS8*** | 0.430 | 0.112 | 14.723 | 1 | 0.125x10^-3^ |
| ***AS15*** | 0.570 | 0.135 | 17.896 | 1 | 0.233x10^-4^ |
| *Constant* | -5.668 | 0.545 | 107.989 | 1 | 0.270x10^-24^ |
|  | | | | | |
| **21^st^-century model** | | | | | |
| ***Model* goodness of fit** | χ² = 6443.0004; *p*<0.05 | | | | |
| **Variable** | **B** | **SE** | **W** | **DF** | **S** |
| *Y-20th century* | 0.466 | 0.021 | 501.666 | 1 | 0.413x10^-110^ |
| ***Bio15*** | 0.005 | 0.001 | 13.397 | 1 | 0.252x10^-3^ |
| ***Bio12*** | -0.276x10^-3^ | 0.571x10^-4^ | 23.468 | 1 | 0.127x10^-5^ |
| ***Bio5*** | 0.008 | 0.001 | 41.104 | 1 | 0.144x10^-9^ |
| ***Bio7*** | -0.004 | 0.001 | 22.930 | 1 | 0.168x10^-5^ |
| *Slope* | 0.188 | 0.023 | 66.532 | 1 | 0.344x10^-15^ |
| ***DeXS*** | -0.259 | 0.126 | 4.211 | 1 | 0.040 |
| ***MedFWS*** | -1.690 | 0.777 | 4.728 | 1 | 0.030 |
| ***TempGSS*** | 1.363 | 0.270 | 25.479 | 1 | 0.447x10^-6^ |
| ***FAmerica*** | 3.591 | 0.239 | 226.400 | 1 | 0.363x10^-50^ |
| ***FAfrica*** | 0.500 | 0.305 | 2.688 | 1 | 0.101 |
| ***FEurope*** | 4.346 | 0.737 | 34.770 | 1 | 0.371x10^-8^ |
| ***FAsia-Oceania*** | 1.853 | 0.247 | 56.112 | 1 | 0.685x10^-13^ |
| ***SA2*** | 0.295 | 0.083 | 12.514 | 1 | 0.404x10^-3^ |
| ***SA4*** | 0.631 | 0.105 | 36.019 | 1 | 0.195x10^-8^ |
| ***AF2*** | 0.112 | 0.035 | 10.125 | 1 | 0.001 |
| ***AS5*** | 0.748 | 0.138 | 29.352 | 1 | 0.603x10^-7^ |
| ***AS7*** | 0.255 | 0.070 | 13.246 | 1 | 0.273x10^-3^ |
| ***AS8*** | 0.352 | 0.135 | 6.818 | 1 | 0.009 |
| ***AS9*** | 0.430 | 0.100 | 18.342 | 1 | 0.185x10^-4^ |
| ***AS15*** | 0.681 | 0.223 | 9.298 | 1 | 0.002 |
| ***AS19*** | 2.134 | 0.616 | 11.994 | 1 | 0.001 |
| *Constant* | -3.332 | 0.396 | 70.638 | 1 | 0.429x10^-16^ |
|  | | | | | |
| **21^st^-century refined model** | | | | | |
| ***Model* goodness of fit** | χ² = 6932.184; *p*<0.05 | | | | |
| **Variable** | **B** | **SE** | **W** | **DF** | **S** |
| *Y-20th century* | 0.339 | 0.021 | 257.529 | 1 | 0.593x10^-57^ |
| ***Bio12*** | -0.240x10^-3^ | 0.520x10^-4^ | 21.235 | 1 | 0.406x10^-5^ |
| ***Bio5*** | 0.005 | 0.001 | 20.916 | 1 | 0.408x10^-5^ |
| ***Bio6*** | 0.007 | 0.001 | 83.845 | 1 | 0.535x10^-19^ |
| *Class 11-14* | 1.924 | 0.203 | 89.857 | 1 | 0.256x10^-20^ |
| *Class 150* | -1.674 | 0.766 | 4.781 | 1 | 0.029 |
| *Class 220* | 5.099 | 1.387 | 13.523 | 1 | 0.236x10^-3^ |
| *Class 60* | -5.482 | 1.111 | 24.349 | 1 | 0.804x10^-6^ |
| *Class 70* | -1.247 | 0.502 | 6.169 | 1 | 0.013 |
| ***Dist_rail*** | -0.167x10^-5^ | 1.83x10^-6^ | 83.486 | 1 | 0.642x10^-19^ |
| ***Pop_den*** | 0.002 | 1.62x10^-3^ | 116.075 | 1 | 0.458x10^-26^ |
| *Slope* | 0.318 | 0.024 | 168.400 | 1 | 0.165x10^-37^ |
| ***FAmerica*** | 3.888 | 0.251 | 240.155 | 1 | 0.364x10^-53^ |
| ***FAfrica*** | 1.072 | 0.303 | 12.530 | 1 | 0.401x10^-3^ |
| ***FEurope*** | 2.453 | 0.666 | 13.562 | 1 | 0.231x10^-3^ |
| ***FAsia-Oceania*** | 1.777 | 0.240 | 54.843 | 1 | 0.131x10^-12^ |
| ***SA2*** | 0.381 | 0.080 | 22.737 | 1 | 0.186x10^-5^ |
| ***SA4*** | 0.496 | 0.102 | 23.505 | 1 | 0.125x10^-5^ |
| ***AF2*** | 0.111 | 0.035 | 9.867 | 1 | 0.002 |
| ***AS8*** | 0.388 | 0.133 | 8.483 | 1 | 0.004 |
| ***AS19*** | 1.430 | 0.618 | 5.349 | 1 | 0.021 |
| *Constant* | -4.537 | 0.415 | 119.611 | 1 | 0.770x10^-27^ |
