# Supplementary material for: Worldwide dynamic biogeography of zoonotic and anthroponotic dengue
Source: PLoS Negl Trop Dis. 2021 Jun 7;15(6):e0009496. doi: 10.1371/journal.pntd.0009496 (PMC8211191; doi:10.1371/journal.pntd.0009496)
Supplement: S9 Table — AUC: area under the receiver operator characteristic curve; FCT: favorability classification threshold; Kappa: Cohen’s kappa; Sens.: sensitivity; Spec.: specificity; CCR: correct classification rate; Underp.: underprediction rate; Overp.: overprediction rate. (DOCX) [file pntd.0009496.s009.docx]

**S9 Table. Percentage of increase in values of the model predictive-capacity assessment** **(i.e., discrimination and classification performance with respect to disease records of a later period) compared to the descriptive-capacity assessment (i.e., discrimination and classification performance with respect to disease records of the same period).** AUC: area under the receiver operator characteristic curve; FCT: favorability classification threshold; Kappa: Cohen’s kappa; Sens.: sensitivity; Spec.: specificity; CCR: correct classification rate; Underp.: underprediction rate; Overp.: overprediction rate.

|  | MODEL | Records of reference | AUC | FTC | Kappa | Sens. | Spec. | CCR | Underp. | Overp. |
| --- | --- | --- | --- | --- | --- | --- | --- | --- | --- | --- |
| 20^th^  century | Disease | 1970 to 2017 | -1.0 | 0.5 | 58.1 | -14.9 | 6.0 | 4.3 | 86.9 | -72.4 |
|  |  |  |  | 0.2 | 64.6 | -8.7 | 7.1 | 7.1 | 91.3 | -46.0 |
|  | Transmission risk |  | -1.3 | 0.5 | 529 | -21.5 | 5.0 | 2.3 | 84.9 | -75.1 |
|  |  |  |  | 0.2 | 61.8 | -12.7 | 6.3 | 5.5 | 88.6 | -48.0 |
|  | Disease | 2001 to 2017 | -1.2 | 0.5 | 56.6 | -14.4 | 5.5 | 4.0 | 85.8 | -62.9 |
|  |  |  |  | 0.2 | 62.8 | -8.6 | 6.5 | 6.6 | 90.8 | -40.6 |
|  | Transmission risk |  | -1.4 | 0.5 | 51.1 | -21.2 | 4.6 | 2.0 | 83.9 | -64.5 |
|  |  |  |  | 0.2 | 60.1 | -12.5 | 5.8 | 5.1 | 87.7 | -42.3 |
| 21^st^  century | Disease | 2001 to 2019 | 0.0 | 0.5 | 5.5 | -0.5 | 0.8 | 0.7 | 12.1 | -6.5 |
|  |  |  |  | 0.2 | 6.9 | -0.2 | 1.0 | 1.1 | 14.9 | -3.7 |
|  | Transmission risk |  | 0.0 | 0.5 | 4.3 | -1.2 | 0.7 | 0.4 | 12.6 | -6.7 |
|  |  |  |  | 0.2 | 6.6 | -0.3 | 0.9 | 1.0 | 14.5 | -3.9 |
|  | Disease | 2018 and 2019 | -1.5 | 0.5 | -74.9 | 3.1 | -5.2 | -5.0 | -190.0 | 27.7 |
|  |  |  |  | 0.2 | -93.2 | 0.7 | -5.9 | -7.4 | -208.0 | 18.1 |
|  | Transmission risk |  | -2.6 | 0.5 | -74.7 | 1.0 | -4.8 | -4.1 | -114.0 | 30.8 |
|  |  |  |  | 0.2 | -89.9 | 1.3 | -5.7 | -6.9 | -223.0 | 18.9 |
| 21^st^ century  (refined) | Disease | 2001 to 2019 | 0.1 | 0.5 | 5.4 | -0.6 | 0.8 | 0.7 | 12.5 | -6.8 |
|  |  |  |  | 0.2 | 6.7 | -0.2 | 1.0 | 1.0 | 17.0 | -4.1 |
|  | Transmission risk |  | 0.0 | 0.5 | 3.5 | -1.8 | 0.6 | 0.3 | 15.1 | -6.4 |
|  |  |  |  | 0.2 | 6.1 | -0.5 | 0.9 | 0.9 | 16.4 | -4.4 |
|  | Disease | 2018 and 2019 | -1.2 | 0.5 | -71.7 | 4.2 | -5.1 | -4.8 | -243.9 | 28.4 |
|  |  |  |  | 0.2 | -91.3 | 0.8 | -5.9 | -7.2 | -254.6 | 19.4 |
|  | Transmission risk |  | -2.2 | 0.5 | -75.8 | 0.4 | -4.9 | -4.2 | -108.5 | 31.7 |
|  |  |  |  | 0.2 | -86.8 | 1.5 | -5.7 | -6.4 | -196.8 | 21.1 |
