# Supplementary material for: Worldwide dynamic biogeography of zoonotic and anthroponotic dengue
Source: PLoS Negl Trop Dis. 2021 Jun 7;15(6):e0009496. doi: 10.1371/journal.pntd.0009496 (PMC8211191; doi:10.1371/journal.pntd.0009496)
Supplement: S2 Fig — Models with temporally stable variables in the short term were used for building models shown in main text Fig 2. Models with variables subject to potential change over time in the short term were used for building models shown in main text Fig 3. Coast lines source: https://developers.google.com/earth-engine/datasets/catalog/FAO_GAUL_2015_level0. (DOCX) [file pntd.0009496.s011.docx]

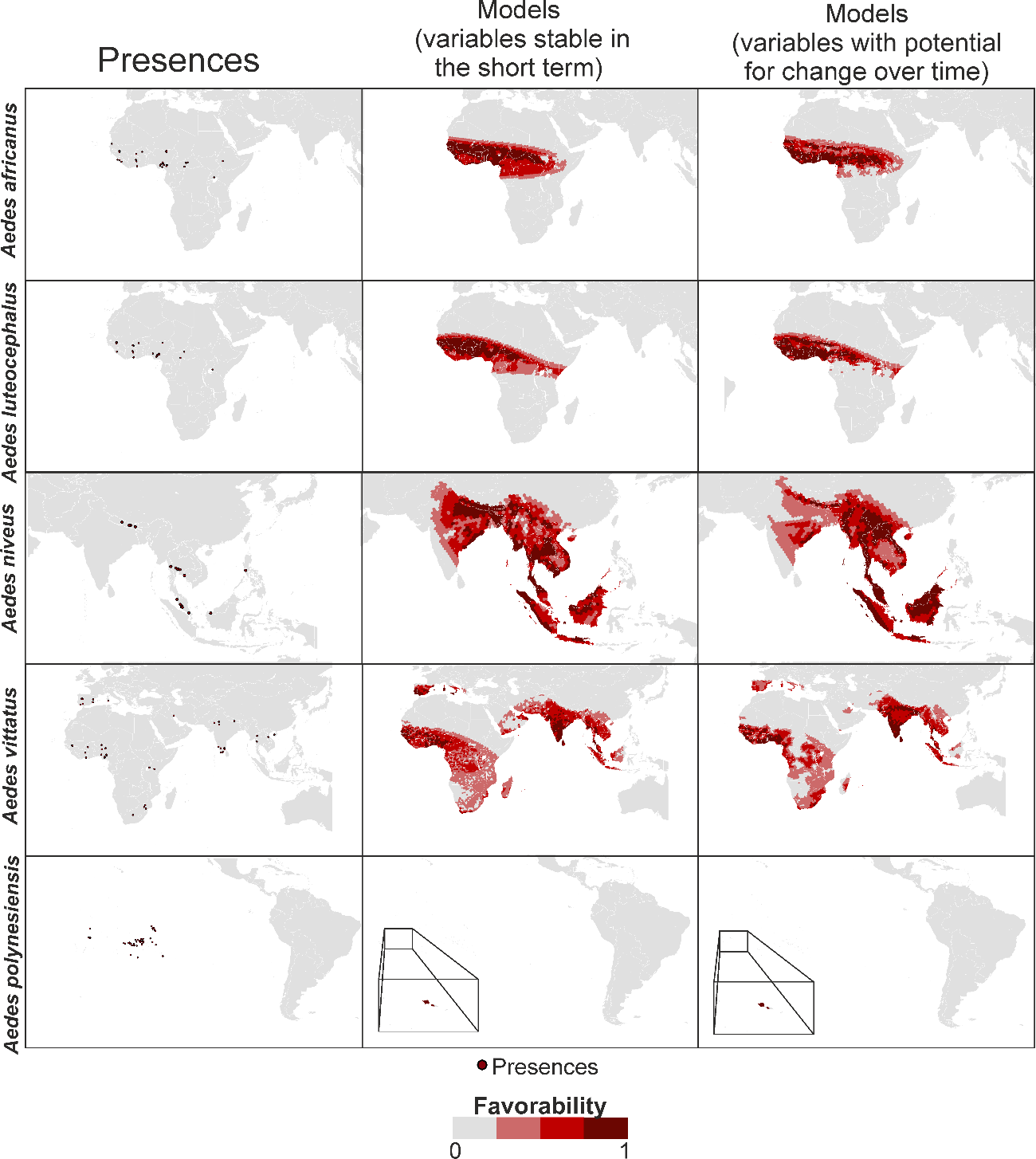


**S2 Fig. Sylvatic-vector presence records and favorability models**. Models with temporally stable variables in the short term were used for building models shown in main text Fig 2. Models with variables subject to potential change over time in the short term were used for building models shown in main text Fig 3. Coast lines source: https://developers.google.com/earth-engine/datasets/catalog/FAO_GAUL_2015_level0.
