# Supplementary material for: Worldwide dynamic biogeography of zoonotic and anthroponotic dengue
Source: PLoS Negl Trop Dis. 2021 Jun 7;15(6):e0009496. doi: 10.1371/journal.pntd.0009496 (PMC8211191; doi:10.1371/journal.pntd.0009496)
Supplement: S3 Fig — Coast lines source: https://developers.google.com/earth-engine/datasets/catalog/FAO_GAUL_2015_level0. (DOCX) [file pntd.0009496.s012.docx]

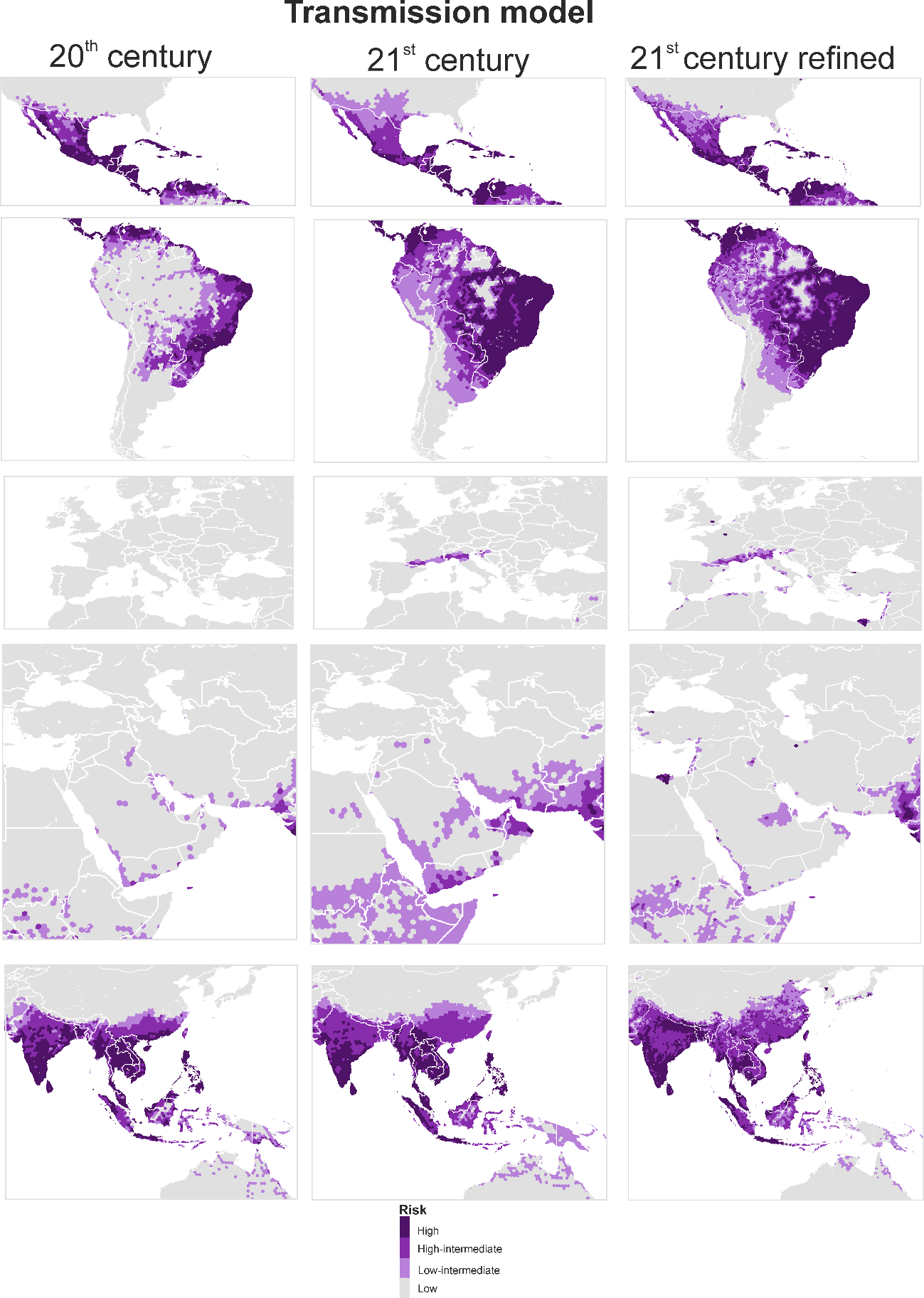


**S3 Fig**. **Zoomed details of dengue transmission-risk models shown in main text Fig 2 (late 20^th^ and 21^st^ century) and 3 (early 21^st^ century refined**). Coast lines source: https://developers.google.com/earth-engine/datasets/catalog/FAO_GAUL_2015_level0.
