# Supplementary material for: Worldwide dynamic biogeography of zoonotic and anthroponotic dengue
Source: PLoS Negl Trop Dis. 2021 Jun 7;15(6):e0009496. doi: 10.1371/journal.pntd.0009496 (PMC8211191; doi:10.1371/journal.pntd.0009496)
Supplement: S4 Fig — The risk of transmission is estimated as the intersection (∩) between favorable conditions for the occurrence of dengue cases and favorable conditions for the presence of vector species. Coast lines source: https://developers.google.com/earth-engine/datasets/catalog/FAO_GAUL_2015_level0. (DOCX) [file pntd.0009496.s013.docx]

**
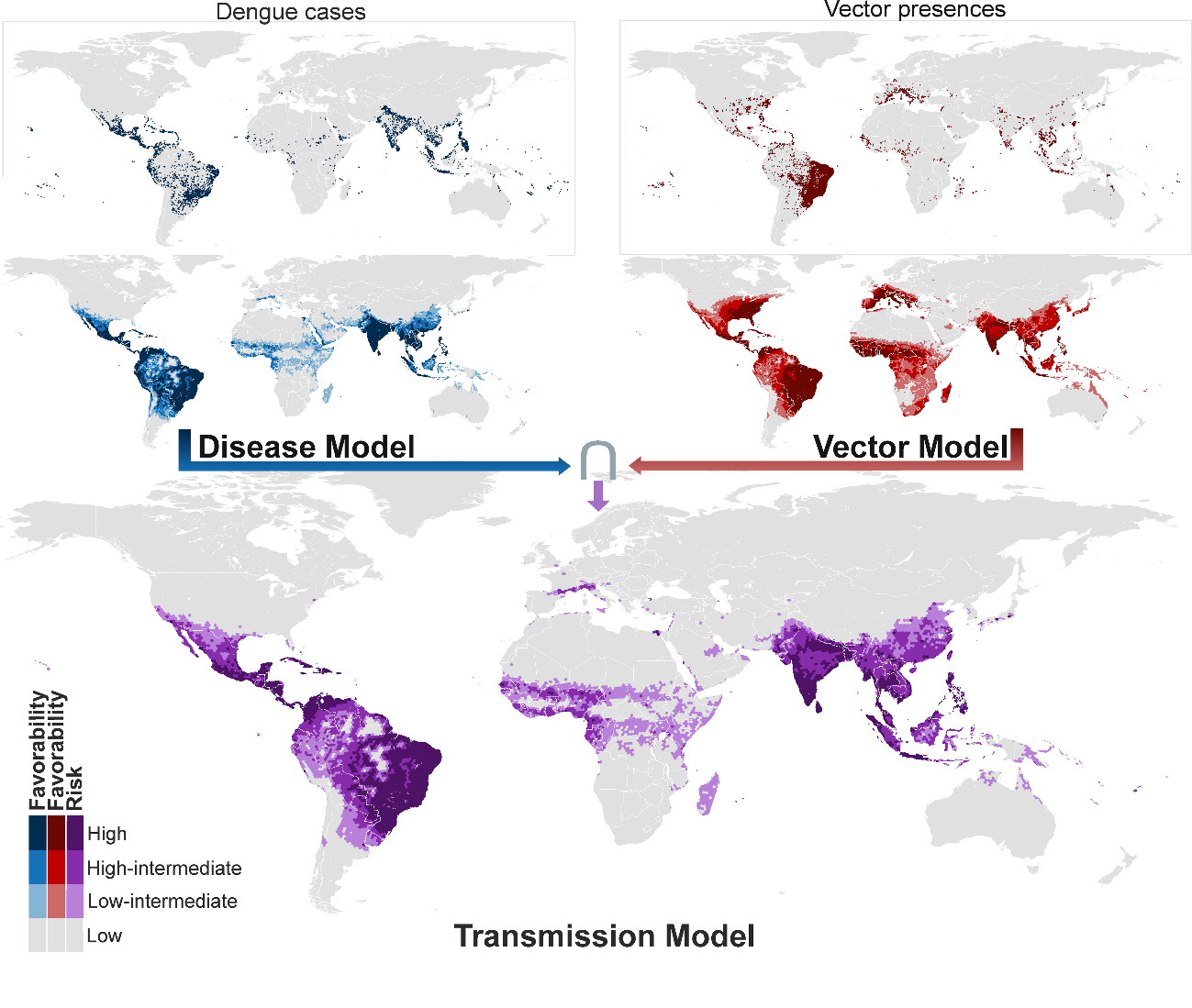
**

**S4 Fig. Pre-downscaling refined global disease, vector, and transmission-risk models for the early 21^st^ century.** The risk of transmission is estimated as the intersection (ꓵ) between favorable conditions for the occurrence of dengue cases and favorable conditions for the presence of vector species. Coast lines source: https://developers.google.com/earth-engine/datasets/catalog/FAO_GAUL_2015_level0.
