# Supplementary material for: Worldwide dynamic biogeography of zoonotic and anthroponotic dengue
Source: PLoS Negl Trop Dis. 2021 Jun 7;15(6):e0009496. doi: 10.1371/journal.pntd.0009496 (PMC8211191; doi:10.1371/journal.pntd.0009496)
Supplement: S6 Fig — Violet rectangles: chorotypes significantly related to the distribution of the late 20th-century dengue cases according to a forward-stepwise logistic regression. Green rectangles: chorotypes significantly related only to the distribution of the 21st-century cases. Chorotypes that were finally included in disease models are highlighted with a violet asterisk for the late 20th century, and with a green asterisk for the 21st century. African chorotype names are coded as AF1 to AF13. Coast lines source: https://developers.google.com/earth-engine/datasets/catalog/FAO_GAUL_2015_level0. (DOCX) [file pntd.0009496.s015.docx]

**
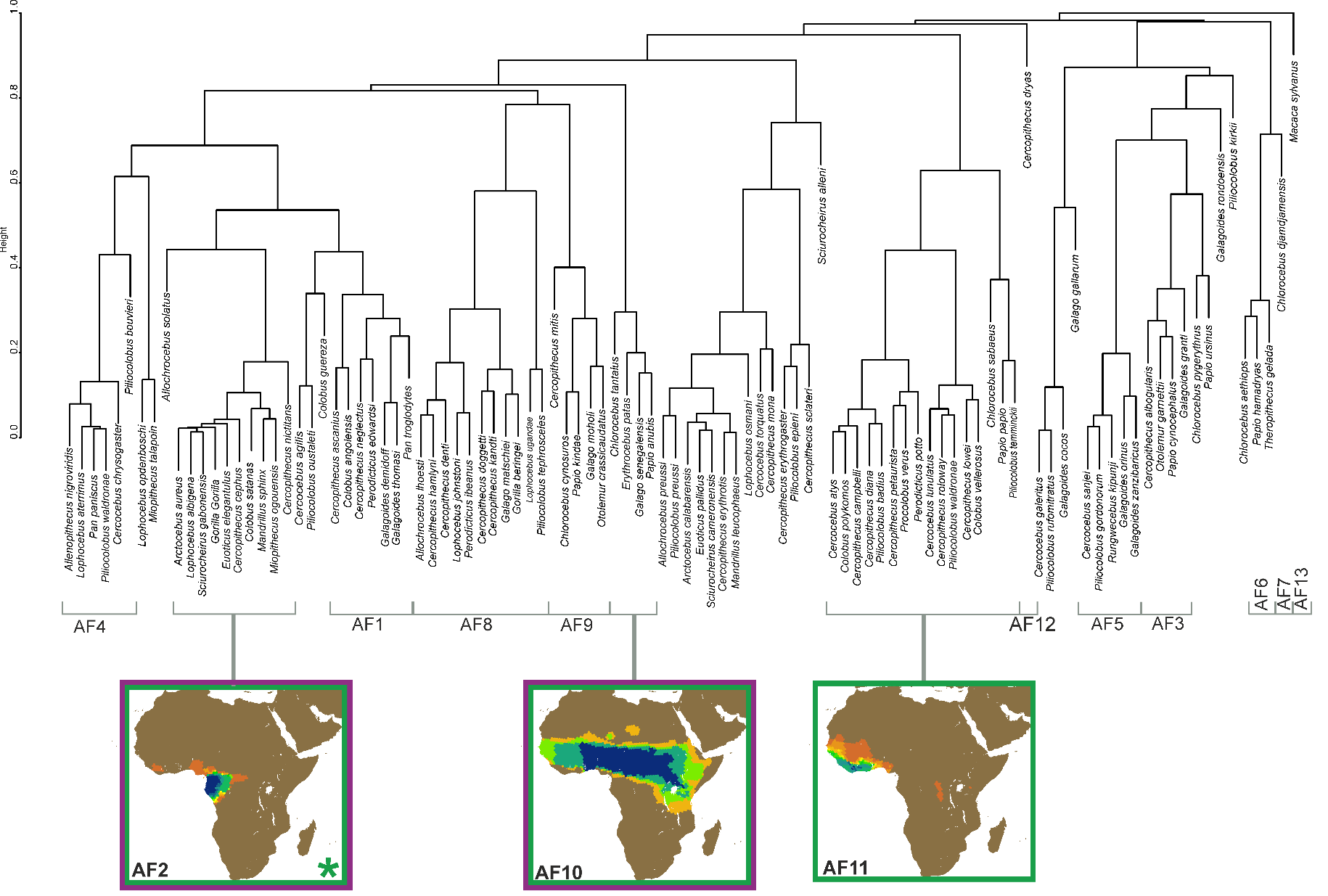
**

**S6 Fig. Classification dendrograms of primate distributions in Africa.**Violet rectangles: chorotypes significantly related to the distribution of the late 20^th^-century dengue cases according to a forward-stepwise logistic regression. Green rectangles: chorotypes significantly related only to the distribution of the 21^st^-century cases. Chorotypes that were finally included in disease models are highlighted with a violet asterisk for the late 20^th^ century, and with a green asterisk for the 21^st^ century. African chorotype names are coded as AF1 to AF13. Coast lines source: https://developers.google.com/earth-engine/datasets/catalog/FAO_GAUL_2015_level0.
