# Supplementary material for: Worldwide dynamic biogeography of zoonotic and anthroponotic dengue
Source: PLoS Negl Trop Dis. 2021 Jun 7;15(6):e0009496. doi: 10.1371/journal.pntd.0009496 (PMC8211191; doi:10.1371/journal.pntd.0009496)
Supplement: S8 Fig — Early 21st-century disease and transmission-risk models in the Indian peninsula (A) and South America (B). These models were calibrated according to human-dengue cases from the late 21st century (Fig 3). The locations of dengue cases recorded in the early 21st century and from 2018 to 2019 are shown in order to illustrate the predictive capacity of these models. Coast lines source: https://developers.google.com/earth-engine/datasets/catalog/FAO_GAUL_2015_level0. (DOCX) [file pntd.0009496.s017.docx]

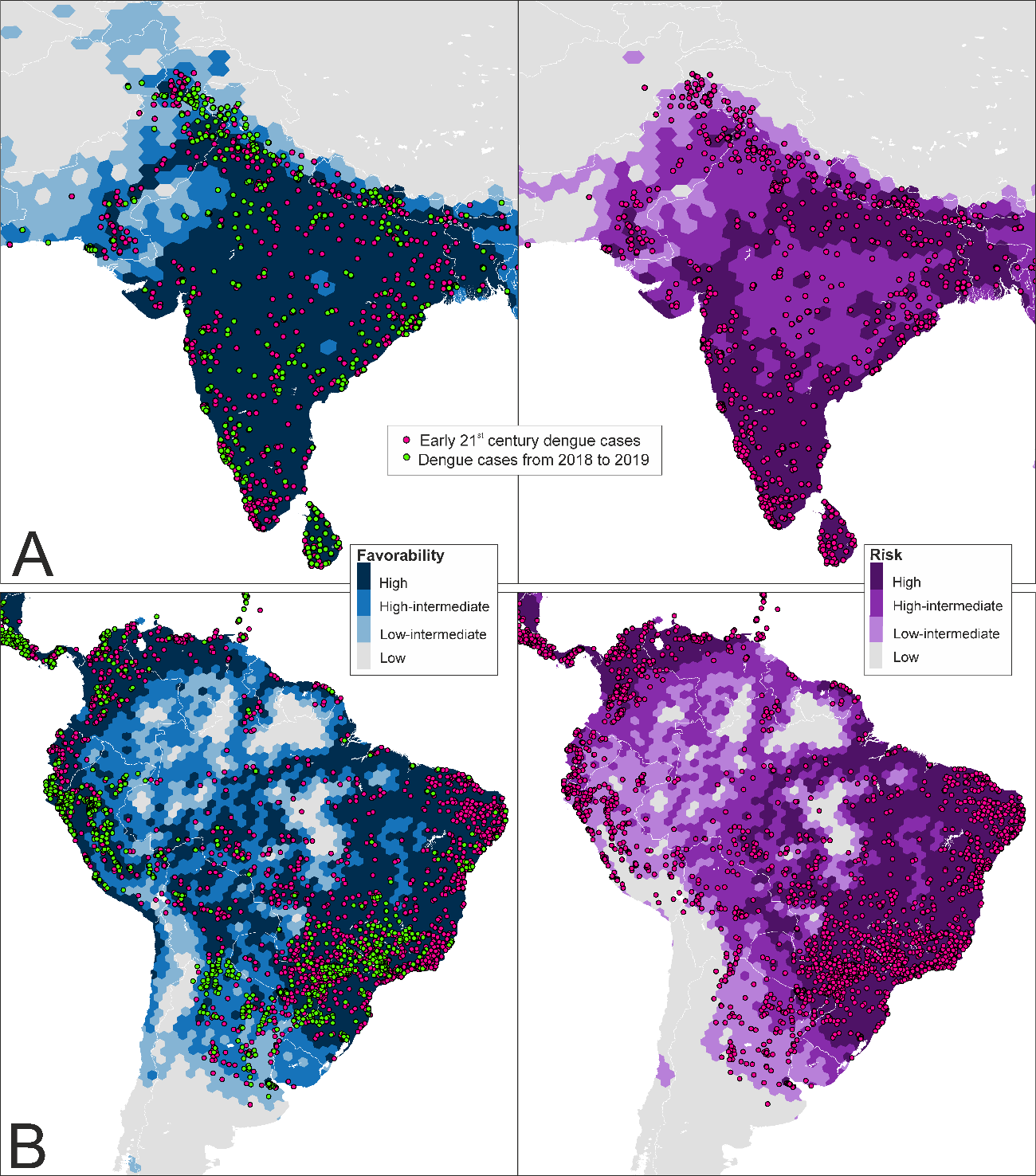


**S8 Fig.** **Early 21^st^ century** **disease and transmission-risk models in the Indian peninsula (A) and South America (B).** These models were calibrated according to human-dengue cases from the late 21^st^ century (Fig 3). The locations of dengue cases recorded in the early 21^st^ century and from 2018 to 2019 are shown in order to illustrate the predictive capacity of these models. Coast lines source: https://developers.google.com/earth-engine/datasets/catalog/FAO_GAUL_2015_level0.
