# Supplementary material for: Worldwide dynamic biogeography of zoonotic and anthroponotic dengue
Source: PLoS Negl Trop Dis. 2021 Jun 7;15(6):e0009496. doi: 10.1371/journal.pntd.0009496 (PMC8211191; doi:10.1371/journal.pntd.0009496)
Supplement: Attachment — Submitted filename: Response to reviewers_PNTD-D-20-01917.DOCX [file pntd.0009496.s018.DOCX]

Dear editor,

We have found in the reviews a very constructive feedback that has helped us to enhance the manuscript by correcting mistakes and improving explanations. The most relevant changes were:

- We have reorganized the abstract.
- We have explained how the original disease-case record was processed and completed.
- We have reinforced justifications for the time periods considered.
- We have given details on methodological decisions that were incomplete or weakly justified.
- We have highlighted the relevance of the spatio-temporal autocorrelation as a way to delimit the spatio-temporal context to which the models are referred.
- We have cleaned the use of “risk” to avoid confusions.
- We have included a paragraph to indicate our model’s limitations.
- We have improved tables, figures and supplementary materials following the reviewer’s advices.
- We have submitted the manuscript to professional English-language revision, and adapted the style to American English.

Please, find below the comments of all reviewers arranged as we have found them in the revision letter, followed by our comments and responses which, just as this paragraph, were highlighted in blue.

**Methods**


**Reviewer #1**: It is not clear how the model was validated on independent data. The authors draw an arbitrary line at the year 2000 and then define each dataset on either side of this as acceptable to validate results against. Is there a previous method to justify this selection or are the authors biasing their results by making this choice? This choice of validation method needs further justification and it needs to be clearer in their methods and results on how this was done.

We assume that dealing with temporal thresholds is always a challenge. Time is a continuous dimension, and the spread of *Aedes* mosquitoes and disease cases during the 20^th^ and 21^st^ centuries was also gradual. That is why, as mentioned in the manuscript (lines 131-135), we needed to set an arbitrary temporal threshold. Anyway, we observed that a threshold in 2000 (i.e. probably a psychologically meaningful date) reflects relevant geographic milestones in the spread of dengue, such as the contemporary record of autochthonous *Aedes*-born disease transmission in Europe and Japan. It is also in the middle of a gradual historical and socioeconomic process that led to the current state of globalization (which is key for understanding the *Aedes* spread). In the new version (lines 135-141), we have added that the bases of the current globalization of international movements were established in the last decade of the 20^th^ century (e.g. with the fall of the Iron Curtain, the advent of the Internet, and the start of low-cost flights); and their full potential was reached just after the start of the 21^st^ century (e.g. with the opening of international borders, the widespread access to the Internet and cell phones, and the online travel booking generalization). In fact, from 1970 to 2000, global exports nearly doubled to approximately a quarter of global GDP. Anyway, model validation consists on testing the fit of model outputs with respect to observations that were not considered in the model training process. And, what is more relevant in our case, the assessment of prediction-capacity consists on using, for model validation, observations regarded to a future period with respect to the model-training data set. Thus, a model predictive capacity can be validated whichever the time limit chosen, on condition that the validation data set is restricted to the period after that limit.

Line 121: Remove first sentence. The authors just need to state they used a worldwide grid for the reader to understand the study area is global.

Done.

Lines 123-127: The downscaling methodology reference needs to be included here and preferably only mentioned once in the manuscript.

The reference to downscaling has been removed from the “Study area and time period” section.

Line 130: Not including pre 1970 records is a limitation placed on results.
We agree that, again, 1970 is an arbitrary limit that imposes limitations that are now mentioned in the Discussion section (see lines 628-659). We chose a 30-years period for representing the dengue-based sanitary, historical and socio-economic scenario of the late 20^th^ century. It was an already technological time, well after the World Postwar II. Some milestones regarding the fight against arboviral diseases occurred around 1970, such as the re-infestation of Latin America with *Ae. aegypti*, main transmitter of the dengue virus, after 50 years of eradication efforts. The use of DDT was suspended in the late 1960s in several countries of the Americas due to resistances. We have added this information to the revised manuscript (lines 127-131). We also follow the reviewer’s advice below to replace "20^th^ century" with "late 20^th^ century" throughout the manuscript.

Why were models updated by adding variables of risk? What distribution of risk?
We have replaced “the distribution of risk” with “the distribution of the modelled item (i.e. vector presences and dengue cases)” (lines 157-158). Also, we have detailed that the rationale for variable addition is that, when animal and pathogen species spread, their distribution at a given moment is influenced by (1) the inertia of previous situations (i.e. temporal autocorrelation), here represented by the late 20^th^-century model; and (2) by a multifactorial set of new drivers potentially favouring the spread (i.e. spatial autocorrelation, environmental and socio-economic factors) (lines 160-163).

Lines 301-303. Why was the lowest favourability value selected for the dengue transmission risk model? This may have resulted in major cities in northern Europe and southern Australia and New Zealand singled out as a risk of transmission, whereas areas throughout these regions were not. Areas surrounding major cities should be just as suitable, as human movement will spread the disease into surrounding areas which, as indicated in Europe in particularly, has a high probable for vector presence. Whereas the Californian San Fernando Valley, a very dry and cold area currently, is predicted to increase in risk over large areas. As such I do not think the transmission model in Figure 3 truly represents the risk of transmission, especially considering the model supposedly takes into consideration all human settlements and authors should justify their choice of selecting the lowest favourability value.

We have re-written, for clarity improvement, the sentence where we justify the use of the lowest favourability value (lines 328-337). In the combination of distribution models, the fuzzy intersection is used when these models represent favourable conditions according to limiting factors (i.e. factors that describe imperative conditions for the modelled item to be present) [Romero et al. (2016) Ecography 39: 561-571 (74)]. The fuzzy intersection consists in selecting, for every location, the lowest favourability value among those obtained in the different models (66). In this study, the transmission-risk model reflects that, for the pathogen to be transmitted in a given location, two elements, acting as limiting factors, must coincide in the area: (1) suitable environmental conditions for disease cases to occur; and (2) suitable conditions for the presence of vectors. This intersection approach has been used before to reflect the simultaneous need of suitable environments and mammal assemblages for the zoonotic transmission of the Ebola virus to humans [Olivero et al. (2017) Mammal Review 47: 24-37 (18)].

The reviewer is right when feeling skeptical respect to some scattered points in Northern Europe and Southern Australia. This is, indeed, an artifact, and thus has been outlined in the new version where the limits of our models are discussed (lines 628-659). However, the fuzzy intersection was not responsible for these points in the map. In the new supplementary material S4 Fig., it can be seen that the transmission model, in its original spatial resolution, did not highlight favourable conditions in these areas. So, the bizarre points have been produced in the downscaling process. In the new version, we have excluded from downscalled outputs all favourable areas that were not also described by the model before downscaling (lines 370-372).

Finally, given the motivations and mathematical meaning of the fuzzy intersection, there is no reason for attributing to this operation the model predictions in San Fernando Valley, California. The predictions of the late 20^th^-century model (Fig. 2) already anticipated the possibility of a favourability increase next to the USA borders with Mexico. The 21^st^-century case records have confirmed this trend (see Fig. 2). Now, the 21^st^-century model reinforces the predictions of a risk increase in the area, which seems to us consistent with the observed trends, despite the dry conditions of the area (Figs. 2 and 3), where *Aedes aegypti* is now keeping stable populations (https://www.dailynews.com/2016/09/07/zika-prone-mosquitoes-found-in-san-fernando-valley/).

Vector favourability

It should be made clearer by the authors that this model does not accurately estimate the favourability for vectors, as it is biased by contemporary vector records. Authors should be careful of making statements about changes in vector favourability, or predictions of future favourability such as Lines 384-385 “models show both species expanded their favourable areas”. It is highly likely that these areas were always favourable for the presence of the species, however, the mosquito had either been eliminated from them previously (*Aedes aegypti* DDT interventions) or as in the case of Europe and Ae. albopictus, had been newly introduced. In both these cases the spread of populations is not related to changes in favourability in environmental characteristics and the authors need to acknowledge this limitation in their model framework.

We fully agree with the reviewer’s comment. We cannot strongly claim that these species are spreading because of changes in environmental favourability. Our models are designed for specific contexts in the temporal dimension, and so the spatial situation of a species can influence its chances to reach or not to reach new areas. We have specified in the new version of the manuscript (lines 393; 396; 429 and so on) that, in some areas, there is increase in favourable conditions which are defined by both environmental variables and spatial constraints. The spatial factor (see the trend surface approach, now modified in lines 230-240) has been very important in all models. This factor could distinguish areas with similar environmental conditions but different chances to be reached by a spreading species, because only in some cases the species is close to the area (i.e. spatial autocorrelation), for example in North and South America; or because it has been introduced/reintroduced in recent times, for example in Europe. In the case of *Aedes alpobictus*, besides, cases of adaptation to new areas could be taking place (see line 678-685), generating environmentally suitable areas in previously unexpected places. More emphasis is now given to the meaning of the spatial factor through the manuscript.

**Reviewer #2:** See below

**Reviewer #3:** The objectives of the study are clearly stated, that is, to investigate the fine-scale geographic changes in transmission risk since the late 20^th^ century, and take into account for the first time the potential contribution that primate biogeography and sylvatic vectors make to increasing the risk of the disease.
The study design and the population are appropriate. Statistical analysis used to support conclusions were correct. Although the methodology is appropriate, the categorization of favourability values need to be clarified.

We have added in the text (lines 343-346) that F = 0.2 and F = 0.8 match approximately the inflection points in the logistic favourability function; while F = 0.5 is the threshold above which the transmission probability defined by spatial and environmental factors is higher than the random transmission probability [Acevedo & Real (2012) Naturwissenschaften 99: 515-522] [51]. Other corrections regarding to this subject have been made (see below).

--------------------

**Results**


-Does the analysis presented match the analysis plan?

-Are the results clearly and completely presented?

-Are the figures (Tables, Images) of sufficient quality for clarity?

**Reviewer #1:**

Line 365: S1 Fig A does not exist.

Really, there is no Fig. A in S1. “A” was the first letter in the following sentence, and intended to be the genus of *A. albopictus*. We wish it is clearer now that we have replaced *A. albopictus* with *Ae. albopictus*.

Line 415: Use of the phrase “favourable areas for dengue have spread” is a more appropriate way to use the favourable index derived in this manuscript. This is due to the necessity of the vector to be present for dengue to be transmitted. Authors should focus on this terminology when using changes in risk predicted by their models. However, it is quite hard to justify an increased risk of disease transmission in small areas like South Australia (Fig 3) which has never had any of the vectors or disease present, and not include large areas of south eastern United States (eg. Mississippi, northern Florida) which has had both Ae. aegypti and Ae. albopictus present historically and epidemics of dengue. The authors need to justify why these historical areas are considered favourable or not in their models, not draw attention to small areas of predicted “favourability”, and if unable to justify these, need to be described as a limitation.

In our above answer to the comments motivated by lines 301-303 (line numbers in the original manuscript), we have recognized that our references to transmission risk in South Australia were mathematical artifacts derived from the model downscaling process. In the new version, we have cleaned the downscaled output from these errors (see lines 370-372). This said, according to our models, some populated areas in Chile, Iran, Iraq and the Maghreb, still free from invasive *Aedes*, possess favourable conditions for transmission; whereas extensive regions of Eastern USA (except for populated areas like Dallas and coastal Florida) are not such much. We have revised some parts of the manuscript to explain better that our models describe favourable conditions according to the environment, but also according to spatial constraints. Our models point to a low transmission risk in Eastern USA because the conditions are not very favourable to the presence of disease cases. The long distance to currently infected areas is a factor decreasing risk level. Anyway, we have written that, there, international travelers should be resolutely advised on mosquito-borne diseases and on the importance of using repellents in endemic areas (see lines 713-718). This means preventing that spatial conditions, which are currently unfavourable, turn into favourable through the virus reintroduction in the area.

Line 418. Use of the work significant in scientific literature means that a statistical analysis was performed, especially in a results section. Please provide results of this analysis or remove this word to describe results.

Although we have deleted “significant” in this sentence, its use was justified by the statistical nature of our models (see lines 374). The significance of every variable in the models was presented in supplementary material S4, S5 and S6 tables. In the new version, we have also included in these table legends Chi-square goodness-of-fit tests for every model.

Using the term “risk” is hard for readers to understand what risk actually means within the context on this manuscript unless it is clearly defined and used consistently through the manuscript for clarity. For instance, the Abstract mentions risk three times – transmission risk, risk of disease and risk factors. Authors needs to be consistent with this language throughout the model.

We have revised the use of “risk” throughout the manuscript, and have retained it only when referred to the risk of dengue transmission.

Figure 3. The manuscript would benefit by ensuring all reference to the transmission model be referred to “dengue transmission risk model”. This will clear up to the reader what risk means in the context of this paper.

We have replaced “transmission model” with “transmission-risk model” throughout the manuscript.

This risk can then be referred to ask either increases in zoonotic and/or anthroponotic dengue transmission risk and the manuscript may benefit by highlighting these changes in different maps.

It is difficult to separate in different maps the zoonotic and the anthroponotic transmission risk, because [we think] zoonotic transmission events usually drive to later anthroponotic transmissions in area. Nevertheless, we propose the areas highlighted in Fig. 4 as those in which zoonotic events might be occurring with high probability.

Line 456: Assessment spelt incorrectly.

Corrected.

Line 458-459: Authors have used a value judgement here instead of stating the results directly. It should be up to the reader to judge the quality of the discrimination capacities as this sounds like confirmation bias.

A reference to Hosmer & Lemeshow [(2000) Applied logistic regression. 2nd ed. New York, USA: John Wiley and Sons] has been added to this sentence. They judged AUC values > 0,8 as “excellent”, and AUC values > 0,9 as “outstanding”.

Line 462: Why is risk used in this sentence? Does it relate to transmission risk? Please state this.

“Risk” has been replaced with “degree of favourability”.

Lines 477-513: All commas in numbers need to be replaced with decimal places.

Replaced.

Line 488: remove the word “got”

Done.

Figure 4 is an informative map and should be improved. Replace commas in numbers. Use one legend on the figure. Light green areas look more yellow than light green. Legend for the light green areas on the figure is uninformative. The description used in the caption should be used instead.

This figure and its legends have been modified. We hope the message is clear now.

**Reviewer #2:** See below

**Reviewer #3:** The results are clearly presented. However, some results need to be clarified or reorganized (the attached file is provide it).
Table and some Figures need to be revised such as the map captions and figure captions.

Thank you very much. The tables and figures have been modified (see below).

--------------------

**Conclusions**


-Are the conclusions supported by the data presented?

-Are the limitations of analysis clearly described?

-Do the authors discuss how these data can be helpful to advance our understanding of the topic under study?

-Is public health relevance addressed?

**Reviewer #1:** There are no conclusions. This is a flaw in the manuscript.

Given that our conclusions are stated through the main text (see reviewer #3), we think that including a conclusion section at the end of the manuscript would distract from the message we outline in the final paragraph.

**Reviewer #2:** See below

**Reviewer #3:** The conclusion is stated but the limitations are not provided
Limitations of our analysis have been added in the new version (see lines 628-659).

--------------------

**Editorial and Data Presentation Modifications?**

Use this section for editorial suggestions as well as relatively minor modifications of existing data that would enhance clarity. If the only modifications needed are minor and/or editorial, you may wish to recommend “Minor Revision” or “Accept”.

**Reviewer #1:** Throughout the manuscript:

Aedes should be correctly abbreviated from A. to Ae.

Modified.

Authors should consider the use of “Results predict” or “Our results suggest” instead of “We anticipate”.

We have replaced “anticipate” as suggested throughout the manuscript.

Unless absolutely necessary to emphasise something, references are preferably used at the end of sentences.

Very often, sentences were constructed on information derived from multiple sources. We think that accumulating the references in the end would imply losing the origin of every statement.

What does quantitatively and qualitatively mean with respect to dengue cycles? The authors need to clearly define what they mean with this terminology.

We have eliminated this terminology, and have explained instead that sylvatic dengue cycles contribute a small percentage to explaining the global extent of the human case record, but could be meaningful in sanitary terms in some tropical areas (lines 772-774).

**Abstract**

Line 13: Remove “successfully”.

Removed.

Lines 26: “distribution” not “distributions”.

Corrected.

Line 34: Suggest using “novel” instead of “new”.

Modified.

Lines 29-30: Remove “the” from “in the dengue-transmission risk”.

Removed.

**Introduction**

Combine paragraphs 2 and 3 into one paragraph for brevity.

Done.

Line 75: Transmitted spelt incorrectly.

Corrected.

Line 89 and elsewhere. Re-emergence spelt incorrectly.

Corrected.

Line 104: Remove “that”.

Removed.

Lines 107-108: Remove “This is the objective of the present study”. This is evident.

Removed.

**Reviewer #2:** See below

**Reviewer #3:** MAIN POINTS:

Lines 3-23: Based on submission guidelines (Manuscript organization),
“The Abstract is conceptually divided into the following three sections with these headings: Background, Methodology/Principal Findings, and Conclusions/Significance.”
I would suggest reorganizing the abstract following these guidelines.

We have reorganized the abstract, so that the limit between background/objectives and methodological information should be clearer now. Strictly, the results are represented by the fact that we get models that are successfully validated. All the later contents of the abstract corresponds to the conclusions and significance of these models.

Line 172: …” Variables with correlation coefficients >0.8 were not allowed…”.
How did you choose 0.8?

We have reorganized the explanation on how multicollinearity was minimized, first through the application of a forward-backward stepwise approach in the variable selection, and later by avoiding high correlations between variables in the model (lines 183-188). Although 0.8 is an arbitrary threshold for “high correlations”, it was simply used as a complement for the more robust criterion applied before. A reference showing precedents in this decision has been provided.

Lines 179-183, Fig 1: It is hard to distinguish between Bottom-left and Bottom-right. Please provide a more detailed explanation!

We have deleted “top”, “bottom-left” and “bottom-right”. Also, the figure legend has been implemented with a more detailed description of the methodological approach.

Lines 304-308: You stated that “Favourability values were finally reinterpreted as transmission-risk values following this scale: high favourability (i.e. F ≥0.8 [69]) was referred as high transmission risk; favourability values between ≥0.5 and 0.8 were referred as intermediate-high risk; … and low favourability (i.e. F < 0.2 [69]) was referred as low transmission risk.

The reference that you refer to, that is, Numoz et all, (2005) mentioned that they classified favourability into only three categories as follows:

“If the predicted favourability was higher than 0.8, which means that the odds are more than 4:1 favourable to the species, the square was considered as favourable. Areas with a favourability value lower than 0.2 (odds less than 1:4) were considered unfavourable to the species. The remaining squares were considered as intermediate favourability areas.”
How did you categorize intermediate-high risk and intermediate-low risk? Please provide some more explanations!

If you follow this reference, line 305 should be … high favourability (i.e. F > 0.8 [69]) was referred as high transmission risk (without “equal” sign).

The reviewer is right. This is the way we applied the thresholds. We have modified it in the text. In order to justify all thresholds, we have also added in the text (lines 343-346) that F = 0.2 and F = 0.8 match approximately the inflection points in the logistic favourability function; while F = 0.5 is the threshold above with the transmission probability defined by spatial and environmental factors is higher than the random transmission probability [Acevedo & Real (2012) Naturwissenschaften 99: 515-522 (51)].

Line 306: “favourability values between ≥0.5 and 0.8” Should not be “0.5 ≤ F < 0.8”?

Line 307: “between ≥0.2 and 0.5” Should not be “0.2 ≤ F < 0.5”?

We have revised the text accordingly.

Line 460: CCR ranged between 0.771-0.884.

Do you mean “ CCR ranged between 0.771 and 0.874?

Yes. We have included "and".

Lines 460-462: It is stated that “ Kappa values were >0.220 in all the 20^th^-century models, and >0.350 (never <0.500 in the disease and transmission models) in the 21^st^-century models.

What do you want to emphasize by including this sentence (never <0.500 in the disease and transmission models)…? My suggestion is as follows: Kappa values ranged between 0.228 and 0.252 in the 20^th^-century models and ranged between 0.352 and 0.533 in the 21^st^-century models.

We have followed the reviewer’s advice (lines 507-509). Besides, we have clarified the sentence under quotation marks, writing “Nevertheless, in the disease models and transmission-risk models for the 21^st^ century, Kappa was always ≥0.518.”

Lines 467-468:…., the CCR values decreased by about 13%,…
How did you get 13%?

The text has been improved (lines 514-518): Compared to the 0.5-favourability threshold, when a 0.2 threshold was adopted, the CCR values of all models decreased by an average 12.67% (SD=3.85), and kappa values did it by an average 31.04% (SD=4.90), which is related to the average 14.91% (SD=4.25) decrease observed in the specificity values.

Line 498: The 21^st^ century (2001-2017) models … record (Table 2).
However, in Table 2 it is stated 2000 to 2019. Which one is correct?

Corrected. The predictive capacity of 21^st^ century models was assessed in relation to dengue-case records of the period 2001-2019, and also in relation to those of the period 2018-2019 (Table 2). The paragraph has been revised to improve clarity (lines 547-554).

Line 515: A total of 51 chorotypes were detected: 24 chorotypes in Asia,…
Fig S4 is unclear. Asian chorotype names are coded as AS1 to AS24. However, we can only see clearly 12 codes among 24 code names. Please make it clearer!

Modified. Thank you for your suggestion.

Lines 516-519: “Two Asian chorotypes form part of the 20^th^-century disease model,….”
Is that correct that only two Asian chorotypes from part of the 20^th^-century disease model (AS8 and AS15)? What about AS7, AS9, and AS19? In S4 Fig you stated that “Violet rectangles: chorotypes characterizing the distribution of the 20^th^-century dengue cases….”

It is true that S5-S7 figures (before S4-S6 figures) were confusing. Now we hope it is clear that chorotypes inside the violet and green rectangles are those significantly related, according to a forward-stepwise logistic regression, to the distribution of dengue cases of the 20^th^ and the 21^st^ centuries, respectively. Also, we have distinguished between violet and green asterisks to indicate which chorotypes were finally included in the respective disease models.

Lines 523-525: “…the 21^st^ -century model also comprised, from Asia: Macaca in chorotype AS5; Hylobates, Presbytis, Symphalangus and Nycticebus in chorotype AS7, Loris, Semnopithecus and Macaca in chorotype AS9, and Carlito in chorotype AS19;”. Is that correct? Should not be only AS5 included?

The Asian chorotypes that were finally included in the 21^st^-century disease model were four, as written in the main text: AS5, AS7, AS9 and AS19. We wish it be clear now, as S5 Fig. has been corrected showing violet and green asterisks as explained above.

Lines 525-527: “from Africa: … in chorotype AF2”. Should not be included in the 20^th^ century?

Chorotype AF2 was included in the 21^st^-century disease model. No African chorotype was directly implied in the 20^th^-century model. Now that the asterisk is green in S6 Fig., we hope it is clearer.

Lines 514-528: Overall, I found the paragraph to be confusing. I would suggest that first explain the chrotypes in Asia (S4 Fig), then chrotypes in Africa (S5 Fig) and America (S6 Fig) of the 20^th^-century disease model followed by the 21^st^-century disease model. Please reorganized!

We have reorganized the paragraph according to the reviewer suggestion.

Lines 529-530: “Primate chorotypes contributed to explain a maximum of 16.4% of the variation in favourability for the presence of dengue in the 20^th^-century (Fig. 4)”.
I would suggest change (Fig. 4) to be (Fig. 4A) as this sentence only related to Fig. 4A

Modified.

Line 534: “In the early 21^st^-century model, chorotypes contributed a maximum of 9.17% to explain the…”

Do you mean 9.8%? 0.7+9.1 =9.8

The reviewer is right. We have corrected the number.

Lines 534-535: “the early 21^st^-century model, chorotypes contributed a maximum of 9.17% to explain the variation in favourability, although only a 0.7% could be exclusively attributed to them”.

I would suggest adding (Fig. 4B) to the end of this sentence.

Added.

Lines 543-544, Fig.4.: The map captions are too small for all 2 maps. Please make it readable! I would suggest making the maps bigger.

The captions inside the figure have been deleted, and the legend has been modified accordingly.

Lines 551-556: I think Figure captions are too long. I would suggest providing a more detailed explanation regarding Fig. 5 in a paragraph.

This legend has been totally rewritten and reduced. Explanations and implications were already provided in the Discussion.

**MINOR POINTS:**

Line 164: …according to Rao’s score tests, …

I would suggest including a reference

Done: Rao, C. R. (1948) Large sample tests of statistical hypotheses concerning several parameters with applications to problems of estimation. Math. Proc. Camb. Philos. Soc. 44, 50–57 [52].

Line 169: … and Wald tests

I would suggest including a reference

Done: Wald A. (1943) Tests of Statistical Hypotheses Concerning Several Parameters When the Number of Observations is Large. Trans Am Math Soc. 54: 426 [53].

Lines 187, 236: …. georreferenced…

Should not be “georeferenced”?

Corrected.

Line 243: Spell out ECDC when it was first mentioned.

European Centre for Disease Prevention and Control (ECDC)

Done.

Line 247: Spell out PAHO

Pan American Health Organization

Done.

Line 248: Spell out GIDEON

Global Infectious Disease and Epidemiology Network

Done.

Line 264: Spell out IUCN

International Union for Conservation of Nature

Done.

Line 323: Bombi &d’Amen [70] shoul be Bombi & D’Amen [70]

Corrected.

Lines 330-331: “…area under the receiver-operating-characteristic curve (AUC) [71].”
Should not be “…area under the curve receiver-operating-characteristics (AUC- ROC) Curve [71]?

We have rewritten the sentence as follows: area under the “receiver operating characteristic (ROC)” curve (AUC).

Line 638: ….whereas Messina et al. ….”

I would suggest including a reference

Done [9].

Line 647: …. by Messina et al. …”.

I would suggest including a reference

Done [9].

Line 697: ….. zoonotic transmission risk (Fig. 4b).

It should be … (Fig.4B).

Corrected.

--------------------

**Summary and General Comments**

Use this section to provide overall comments, discuss strengths/weaknesses of the study, novelty, significance, general execution and scholarship. You may also include additional comments for the author, including concerns about dual publication, research ethics, or publication ethics. If requesting major revision, please articulate the new experiments that are needed.

**Reviewer #1:** The manuscript “Worldwide dynamic biogeography of zoonotic and anthroponotic dengue” builds several models to predict both the potential distribution of dengue vectors and the risk of dengue transmission across a number of years in the 20^th^ and 21^st^ century. Incorporating sylvatic cycles and a zoonotic component is an important issue and I believe this type of analysis will be important in predicting future emerging infectious disease hotspots. The authors have gone put considerable effort into incorporating these factors into their predictions, especially as sylvatic cycles are related almost entirely on environmental conditions. The manuscript would benefit from an increased focus on these challenges.

The manuscript has merit for publication in PLOS NTD, however, there are a number of issues that will need to be addressed before I believe it will reach the standard required. The manuscript is over 12,000 words and there are many areas where this can be reduced for brevity and clarity. For instance, English may not be the authors first language but many of the statements made throughout the manuscript need to be clarified, removed or simplified as they can be irrelevant to the narrative. Some of the priority issues are listed below.

A major problem with the manuscript is a failure to frame their results correctly in the context of Aedes historical distributions with respect to “favourability” and in particular with *Aedes aegypti* historical distributions. There has been extensive documentation on the distribution of this vector for over 100 years now, and yet the results in this manuscript are biased by the distribution they have chosen (from Kraemer et al 2015) which does not include records of the species at a time before DDT was used to eliminate large populations of this species. This includes areas in North America, South America, Australia and Europe which were clearly “favourable” to the species but not included in this model. As such authors first need to reframe their argument throughout the manuscript in terms of “late 20^th^ century” and “early 21^st^ century and not “20^th^ century” or “21^st^ century”. I noticed this has occurred in some places, including on figures but it is important for consistency that this terminology is used throughout the manuscript. This will have an impact on the way results and discussion are framed.

Linked to this issue is the confidence in which authors state how accurate their results are. No model is perfect, and modellers should be careful when making absolute statements or value judgements about the world or the quality of their results. By not including species distribution records from before 1970 the “favourability” model is already inaccurate as Ae. aegypti distributions were considerably larger before this time, confounding author’s conclusions. As *Ae. aegypti* is the primary vector of dengue, this considerably increased the risk of dengue transmission in the early 20^th^ Century. For example, there were huge epidemics of dengue in Greece (~30,000 cases) and Australia (~100,000 cases) in the 1920/30s which impacted the majority of the population in affected areas. Aedes aegypti distribution is not linked entirely to environmental variables, but also artificial water storage by humans. It was artificial water storage which led to these large outbreaks in the early 20^th^ century in areas of Australia and Greece where rainfall is not high enough for the species to survive on rainfall alone. This is not adequately represented in the model and must be stated as a limitation to conclusions. Furthermore, these areas in Australia are not currently represented as dengue transmission risk in Figures 2, and only barely become represented in Figure 3 and I believe this is a failure of the model.

We have included “late 20^th^ century” and “early 21^st^ century” throughout the manuscript as suggested by the reviewer. In addition, we have provided more detail about the temporal context of our analysis, with explicit references to the “post DDT” situation and to the current globalization of communications (see the second paragraph of “Study area and time period”, in the Methods section). We probably did not explain sufficiently that, with the “trend surface approach” used in our analyses (see lines 230-240), we built models whose predictions on the distribution of transmission risk are expected to be focused on the current historical moment. Through the “trend surface approach”, the models you have seen in the manuscript include the spatial factor as predictor variable, together with potential environmental and sylvatic drivers. We have made more emphasis on this in the corrected version. We have explained that the spatial factor could distinguish areas with similar environmental conditions but different chances to be reached by a spreading species at a given historical moment, which could happen because of spatial autocorrelation (i.e. the species could have or not have nearby populations in some cases); or as a result of recent introductions or reintroductions. Besides, the fact that our models were based on a combination of spatial and environmental variables was not sufficiently highlighted in the results. References to the presence and the strength of the spatial factor in the models are now given throughout the manuscript (e.g. lines 418-420).

Now that we have revised the manuscript, we are aware that we could have be more cautious when describing the success we perceive in our results. We, as biogeographers that find in distribution modelling a strong methodological support, are used to get models that do not work, or that are based on too incomplete information as to represent real situations. Some models are bad, and in these cases the models are not published or must be enhanced. When the reviewer feels that we made “absolute statements or value judgements about the world or the quality of [our] results”, he/she had been probably surprised to read how we qualified our AUC values as “outstanding” (line 504). We were not using our own words. Now we explain that “outstanding” is how Hosmer, D.W. & Lemeshow, S. [(2000) Applied logistic regression. 2nd ed. New York, USA: John Wiley and Sons] qualified AUC values higher than 0.9. We would also like to highlight, however, that we ourselves were happily surprised to see how our models based on case records from the late 20^th^-century produced outputs that matched even better with the case records of the early 21^st^ century. In our opinion, this is an objective way of supporting the predictive capacity or our models. We have tried to express it properly in the manuscript. Anyway, we are also aware that our models have limitations, and have described them in a new paragraph (see lines 628-659).

We have also emphasized, when justifying the predictor variables considered, why socio-economic variables such as human population density, distance to populated places, irrigation and livestock were considered in the vector models and the disease models (see lines 357-361). Not only the availability of potential victims, but also the availability of human-modified environments (such as water points) favourable to the reproduction of urban *Aedes* mosquitoes, motivated the use of these variables. These variables were finally selected by the stepwise procedure to forming part of the models (see S4, S5 and S6 Tables). Anyway, in our new statement of model limitations, we outline the fact that areas that were suitable for dengue transmission in the past might not be detected by our models, which are, instead, focused on the current historical moment (line 628-659).

The reviewer is also right to suppose that most of the authors are not native English speakers. Such is not the case, however, of one of we, who revised the final version carefully. Anyway, the new version has been revised by a professional proofreading company.

**Reviewer #2:** Authors present a worldwide model of dengue transmission risk using updated vector and dengue case data. They included data from the sylvatic vectors of dengue and also chorotypes for non-human primates. The entire modeling is based on fuzzy logic (fuzzy sets) and logistic regressions with stepwise forward-backward model selection, for example, that is how they develop their chorotypes. The study is well written, well referenced and novel. However, they are evaluating the models based on discriminatory statistics without true absence data. A crucial problem on this type of analysis that should be explicitly declared, presented and dealt with, which is currently not the case.

We thank reviewer #2 for their comments. We have added a text with limitations of our models (see lines 628-659). One of these limitations is that our models were based on the presence/absence of occurrence records. There are no true-presence/true-absence data available on vectors and disease cases. One of our ways to deal with this problem was the grid approach, which has filled some (though not all) gaps, as it involved a decrease in spatial resolution compared to the original point data. This reduced to a large extent the proportion of area considered to be free from dengue in the data base. Biases derived from false absences are a main problem when the modelling method is susceptible to overfitting, which is not the case with our approach [83]. In our models’ assessment, specificity values (from 0,580 to 0,891) were lower that sensitivity values (from 0,822 to 0,991), meaning that 11-42% of the “absence” hexagons were predicted to be favourable by the models. Anyway, some bias could occur in poorly sampled regions, for example in Africa, if the areas with disease records did not represent the complete range of environmental conditions in which the dengue is transmitted to humans. So, the outputs and model predictions in Africa should be interpreted with caution, and thus we indicate in the revised version. We have explained this in detail in a paragraph we have added to explain our model limitations (lines 628-640).

See further comments:

Lines 329-336: Authors should provide a better justification of using AUC and the rest of discriminatory statistics based on presence/absence data. Even their own reference (i.e., 71) present important arguments on why those statistics should not be used for model selection. Specifically, absence data is lacking and the use of the entire globe as study area is for sure inflating their statistics, thus, authors should be more careful on the interpretation of their ‘excellent’ predictive power (line 596). This is a very important problem of the entire modeling framework since render their metrics useless. See also: Peterson et al 2008 (doi: 10.1016/j.ecolmodel.2007.11.008) and Jiménez et al 2020 (doi: 10.1111/2041-210X.13479). I will recommend to use instead omission rates (i.e. a performance metric; Galante et al 2017 (doi: 10.1111/ecog.02909), Cobos et al 2019 (doi: 10.7717/peerj.6281) and include in the discussion a section commenting on the difficulties of model selection on species distribution models (Velasco et al 2018 (doi: 10.1016/j.ecoinf.2019.02.005)). Using study areas specific for each vector might also be very useful (Barve et al 2011 doi: 10.1016/j.ecolmodel.2011.02.011).

We know the reference according to which the reviewer affirms that the AUC and the rest of discriminatory statistics based on presence/absence data should not be used for model selection. One of the authors of that reference is also a coauthor of the current manuscript. Lobo et al. [(2008) Global Ecology and Biogeography 17: 145-151] proposed in their paper is that the AUC should not be used for assessing the accuracy of predictive distribution models, but only for providing a measure of discrimination capacity (literally, “the degree to which a species is restricted to a part of the variation range of the modelled predictors, so that presences can be told apart from absences”). This is what we aimed to do with the AUC. Lobo et al. also indicated that models based on items (e.g. species) showing different spatial prevalence should not be compared using the AUC, but we never did it. The other indices employed for model assessment were not focused on discrimination. Instead, kappa, CCR, sensitivity, specificity, over-prediction rate and under-prediction rate were used for assessing the model classification capacity. We agree with the reviewer that the omission rate is a very suitable index for this purpose. In fact, the omission rate is equal to 1 minus the sensitivity, in the same way as the commission rate is equal to 1 minus the specificity. The reviewer can see that both sensitivity and specificity were among the classification indices considered.

In the discussion, we have replaced “excellent predicted power” with “predictive power”. We may have given the impression of being very proud of our models. In reality, we were happily surprised to see how our models based on case records from the late 20^th^-century produced outputs that matched even better with the case records of the early 21^st^ century. In our opinion, this is a robust and objective way of supporting the fact that our models had a very good predictive capacity.

We believe that a discussion on the difficulties of model selection on species distribution models would be superfluous in our manuscript, as our model building was not based on selecting between different models. We produced models through the combination of predictor factors using statistics and machine learning; and later we combined [but not selected between] different models using fuzzy logic operations. In the legend of Fig. 1, we have now deepened in details on our general methodological approach.

We agree with the reviewer that using study areas specific for each vector might be very useful. We got the same effect when applied the trend-surface approach to our models, that is, when we fixed the spatial context of our models according to the consideration of a purely spatial factor (see details in lines 230-240, 637-640). The most powerful predictor in all models resulted to be the spatial factor (and we give more attention to this fact in the revised manuscript). In species like some sylvatic mosquitoes, whose distributions were restricted to a single continent, this trend-surface approach had the effect of contextualizing the influence of predictor variables to the area defined by the spatial factor.

Authors should mention the number of points used for each model. In this line, please mention the number of occurrences available for each vector including the non-urban mosquito points. Also, please mention the total number of dengue cases used from Messina et al and how many more occurrences where added after your updated search. This information should be presented to have a clearer idea of the evaluation phase, for example, how many points where used for evaluating the 21^st^ century model with 2018-2019 cases?

A new appendix (S1 Table) has been added with this information.

It is not clear how did you include information from public health reports that are usually presented in the form of uninformative geographical centroids in big administrative units (e.g., provinces), instead of actual ‘points’; this might be problematic while transferring information to hexagons by adding inaccurate records to different regions.

The reviewer is right. For this reason, we have only worked with the cases that were referred to a precise location (i.e. points), and also with centroids that were referred to polygons whose extension was lower or similar than the size of the hexagons. In the new version, we have given details on this subject (lines 266-268).

Line 123: Please specify that the first number corresponds to grid cells and the second number to the area represented by the hexagons.

Done.

It makes sense that this approach avoids autocorrelation, however it is not clear if the post-calibration downscaling might introduce another source of uncertainty in your interpretations.

Any model projection will produce a new source of uncertainty. In the case of downscaling, the difference between pre- and post-downscalling hexagon sizes was lower than the maximum recommended by Bombi & D’Amen [(2012) J Biogeogr. 39: 640-651], in order not to lose the ecological meaning of the original model. This was already explained in the original version of the manuscript. Anyway, we have detected a small artifact caused by downscaling, which pointed high risk of dengue transmission in highly populated cities outside the area considered to be at risk by the original model. This artifact has been corrected and explained (see line 370-372, 653-659).

Line 263: Eliminate word ‘primates’

Done.

Lines 199-202: Please make it clear which variables are used for which model in the supplementary material, so readers can refer only to the table to recognize variable application in just one section of the manuscript. This is a comment that should be done considering the refinement of the model (lines 310-318), because according to the main text, for example, livestock density was used for the vector but not for the disease models.

We have modified the S3 Table, indicating which variables are used only in specific models.

Lines 317-318: Was the inclusion of the new set of variables a source of multicollinearity on your models? Were they assessed for collinearity with the other variables?

Yes. Model enhancing was performed through the development of new disease and vector models (we think it can be understood by default that they were subjected to the same method and controls), on the basis of an expanded set of predictors.

Rectangle colors in Supplementary figure number five and six are red instead of violet as mentioned in the legend, please correct accordingly. Names of non-human primate species in Supplementary figure 6 are almost unreadable. Please make sure the figures have enough resolution.

Modified.

Lines 304-308: Please mention that these thresholds are arbitrarily selected and not possess any empirical evidence to reflect either transmission risk category. It is useful for interpretation and should be kept, just be explicit in the way it is working.

New and more specific reasons why these thresholds were chosen have been included in the text (lines 338-346).

Line 635: Please add the name of the algorithm used in this manuscript between parentheses.

Done.

I would like to suggest building a figure similar to Figure 5 using the models of the 21^st^-century. It can be presented in the same figure 5 or as a supplementary material.

Done (S8 Fig.).

**Reviewer #3:** The paper describes the potential contribution of primate biogeography and of sylvatic vectors in increasing the risk of dengue. This research aims to build a map that quantified the current level of dengue transmission risk worldwide by including a zoonotic component as well as temporal stratification in the production of a dengue risk map. The manuscript is generally well-written. However, some points need to be improved and clarified as mentioned above.

Thank you very much. We have improved all the points that have been indicated by the reviewer.
