# Supplementary material for: Worldwide dynamic biogeography of zoonotic and anthroponotic dengue
Source: PLoS Negl Trop Dis. 2021 Jun 7;15(6):e0009496. doi: 10.1371/journal.pntd.0009496 (PMC8211191; doi:10.1371/journal.pntd.0009496)
Supplement: Attachment — Submitted filename: Response to reviewers_PNTD-D-20-01917.docx [file pntd.0009496.s019.docx]

Dear editor,

Thank you for this second round of revisions. We have corrected mistakes and made minor improvements following the advice of the reviewers. The changes were:

- We have corrected grammatical errors.
- We have added a final conclusion paragraph, where we have moved conclusions that were exposed along the discussion section (in order to avoid redundancies).
- We have moved the paragraph describing model limitations as indicated by the reviewers.
- We have added two tables in supporting information.
- We had already submitted the manuscript to a professional English-language revision, and now it has been revised again by one of the authors who is native English speaker.

Please, find below the comments of all reviewers arranged as we have found them in the revision letter, followed by our comments and responses which, just as this paragraph, were highlighted in blue.

**Methods**

**Reviewer #1:** (No Response)

**Reviewer #2:** see below

**Reviewer #3:** Yes, the authors have carefully revised the manuscript.
Thank you very much
--------------------

**Results**

**Reviewer #1:** (No Response)

**Reviewer #2:** see below

**Reviewer #3:** Yes, the authors have carefully revised the manuscript.
Thank you very much
--------------------

**Conclusions**

**Reviewer #1:** The discussion would benefit greatly from a conclusion paragraph. A conclusion paragraph is your chance to have the last word on your findings. Conclusion paragraphs allow you to have the final say on the issues you have raised in your paper, to synthesize your thoughts, to demonstrate the importance of your ideas, and to propel your reader to a new view of the subject. It is also your opportunity to make a good final impression and to end on a positive note.

We have added a final conclusion paragraph, where we have moved conclusions that were exposed along the discussion section (in order to avoid redundancies).

**Reviewer #2:** see below

**Reviewer #3:** Yes, conclusion is clearly stated and the authors have carefully added the limitations in the new version

Thank you very much

--------------------

**Editorial and Data Presentation Modifications?**

Use this section for editorial suggestions as well as relatively minor modifications of existing data that would enhance clarity. If the only modifications needed are minor and/or editorial, you may wish to recommend “Minor Revision” or “Accept”.

**Reviewer #1:** Multiple lines: Replace “transmissions” with “transmission” on lines 6, 77, 109, 114, 767, 769, 787, 799, 810.
Line 8: Remove “therefore”
Line 16: Incorrect spelling of Papua New Guinea.
Line 129: Replace “transmitter” with “vector”
Line 131: Replace “resistances” with “resistance”
Line 133: Sizeable spelt incorrectly
Line 212: Remove the second “the” from the reference title: “The global compendium of the
Aedes aegypti and Ae. albopictus occurrence”
Line 230: Replace “infrastructures” with “infrastructure”
Lines 254-256: Remove second “in the late 20th century” in the sentence
Line 359: Replace “human victims” with “human hosts”
Line 389: “non-human primates” spelt incorrectly
Line 443: Use “Aedes” instead of abbreviation “Ae” when starting a sentence
Line 618: Remove “by that time” from the sentence
Line 620: Remove “broadly”
Line 626: Define the type of risk you are referring to here
Line 628-659: Place the “limitations” paragraph later in the discussion. You want to start the discussion with your most important results.
Line 633: Place “transmission” in front of risk and remove from after dengue.
Line 660: Remove the first phrase about methodological limitations. It is no longer needed here
673: Incorrect spelling of albopictus.
Line 729: Change “South” to “southern”
Line 732: Remove “alone”
Line 756: Informal language “sound alarm bells louder”. Change to “sound a warning”
Line 757-758: Remove hyphens and use commas
Line 766: Remove “the”
Line 772: Remove “being”
Line 797: Rephrase “primate chorotypes was pure” to “pure primate chorotypes”
Line 799: Replace “mapped” with “estimated”
Line 820-823: Rewrite sentence to “This concentration of evidence suggest the need for studies that address the occurrence of dengue sylvatic cycles in the Atlantic forest of Brazil and the Amazon”

Thank you very much for your contributions. All these suggestions were accepted, and the limitation paragraph was moved forward.

**Reviewer #2:** see below

**Reviewer #3:** (No Response)

--------------------

**Summary and General Comments**

**Reviewer #1:** The authors have done a good job in responding to reviewer comments and the paper is almost finished. There is still a number of spelling mistakes, and poor English in the manuscript which will need to be addressed before it can move to publication. The incorrect use of plurals still needs attention and the discussion would benefit from a concluding paragraph.
We have corrected grammatical errors, and the final manuscript has been revised again by one of the authors who is native English speaker. Besides, we have added a final conclusion paragraph.

**Reviewer #2:** Authors have argued favorably to all my comments and I believe the manuscript is now suitable for publication. Regardless, I will insist to include a comment in the point referring to the discriminatory capacity of their models using AUC:

In the paper of Lobo et al., they clearly argue the following: “increasing the geographical extent outside presence environmental domain entails obtaining higher AUC scores” which is clearly something that is happening while using the ‘entire world’ as a calibration area for your modeling effort. Thus, your now ‘outstanding’ discrimination capacity, lies on the inflation of statistics due to including a larger calibration area. The implementation of a ‘trend-surface’ does not fix this problem since you are including it as another co-variate during you model calibration step which is using the entire world for the rest of the predictors as well. Your other statistics based on thresholds (currently justified) are a fair representation of the performance of the models and the majority of your interpretations are based on those results. I will like to encourage you to be explicit on the problems of the AUC to actually invite other authors to avoid using it in the context of lack of true absence data and the inflation of his values due to using calibration areas beyond the accesible area of the studied species (Jiménez & Soberón, 2020: <https://doi.org/10.1111/2041-210X.13479>)

Thank you very much for your comments and contributions. We have written that the “outstanding” discrimination capacity of our model could be a result of the worldwide geographic extent of the calibration area. Anyway, as the invasion by *Aedes* mosquitoes is reaching areas in all continents (transported by humans), sometimes far from the original environmental ranges (as adaptation has been demonstrated), it is difficult to state what is beyond the accesible area of the studied species.

Check typos and old information in Supplementary Table 3: The word should be ADMINISTRATIVE. Also, reference to Supplementary figures in the Primate chorotype section is outdated, it should be Supp. Figs 5-7.	Done.

Lines 418-423. Please consider highlighting the variables mentioned in the main text in the Supplementary Tables S4, S5, and S6. Done.

Lines 440-449: Please consider including a table showing the contribution of your predictors to the favorability models of the dengue sylvatic vectors (as is done with Supp Table 4, 5, or 6). We have included new tables in Supporting Information: S6 and S7 Tables.

Line 534: Favorability instead of favorabity
Corrected.

Line 615: It is not clear what the authors meant with ‘fine-scale’	We have replaced “fine scale” with “high resolution”.

Line 625: The authors are not suggesting any specific management strategies, they are showing potential risk factors.	We have rewritten the sentence, as our study is useful AS A BASIS for suggesting management strategies.

Line 654: Should be ‘still’	Corrected.

Line 659: Refer the reader to the appropriate figures and supplementary material.
Done.

Make sure that Fig S8 is mentioned in the main text.	Done.

**Reviewer #3:** (No Response)

--------------------

PLOS authors have the option to publish the peer review history of their article ([what does this mean?](https://journals.plos.org/plosntds/s/editorial-and-peer-review-process#loc-peer-review-history)). If published, this will include your full peer review and any attached files.

If you choose “no”, your identity will remain anonymous but your review may still be made public.

**Do you want your identity to be public for this peer review?** For information about this choice, including consent withdrawal, please see our [Privacy Policy](https://www.plos.org/privacy-policy).

Reviewer #1: No

Reviewer #2: No

Reviewer #3: No
